# Neuroprotective effect of *Withania somnifera* leaves extract nanoemulsion against penconazole-induced neurotoxicity in albino rats via modulating TGF-β1/Smad2 signaling pathway

**DOI:** 10.1007/s10787-024-01461-8

**Published:** 2024-04-17

**Authors:** Mohamed Abomosallam, Basma M. Hendam, Amr A. Abdallah, Rasha Refaat, Heba Nageh Gad EL-Hak

**Affiliations:** 1 Forensic Medicine and Toxicology Department, Faculty of Veterinary Medicine, Mansoura University, Mansoura, Egypt; 2 Department of Husbandry and Development of Animal Wealth, Faculty of Veterinary Medicine, Mansoura University, Mansoura, Egypt; 3 Central Agricultural Pesticides Laboratory, Agricultural Research Center, Giza, Egypt; 4 Phytochemistry and Plant Systematics Department, National Research Centre, Dokki, Cairo, Egypt; 5 Zoology Department, Faculty of Science, Suez Canal University, 10, Ismailia, 41522 Egypt

**Keywords:** *Withania somnifera*, Nanoemulsion, Penconazole, Neurotoxicity, LC–MS/MS

## Abstract

Penconazole (PEN) is a systemic triazole fungicide used to control various fungal diseases on grapes, stone fruits, cucurbits, and strawberries. Still, it leaves residues on treated crops after collection with many hazardous effects on population including neurotoxicity. *Withania somnifera* leaves extract (WSLE) is known for its memory and brain function enhancing ability. To evoke such action efficiently, WSLE bioactive metabolites are needed to cross the blood–brain barrier, that could limit the availability of such compounds to be localized within the brain. Therefore, in the present study, the association between PEN exposure and neurotoxicity was evaluated, and formulated WSLE nanoemulsion was investigated for improving the permeability of the plant extract across the blood–brain barrier. The rats were divided into five groups (*n* = 6). The control group was administered distilled water, group II was treated with *W. somnifera* leaves extract nanoemulsion** (**WSLE NE), group III received PEN, group IV received PEN and WSLE, and group V received PEN and WSLE NE. All rats were gavaged daily for 6 weeks. Characterization of compounds in WSLE using LC–MS/MS analysis was estimated. Neurobehavioral disorders were evaluated in all groups. Oxidative stress biomarkers, antioxidant enzyme activities, and inflammatory cytokines were measured in brain tissue. Furthermore, the gene expression patterns of GFAP, APP, vimentin, TGF-β1, Smad2 and Bax were measured. Histopathological changes and immunohistochemical expression in the peripheral sciatic nerve and cerebral cortex were evaluated. A total of 91 compounds of different chemo-types were detected and identified in WSLE in both ionization modes. Our data showed behavioral impairment in the PEN-treated group, with significant elevation of oxidative stress biomarkers, proinflammatory cytokines, neuronal damage, and apoptosis. In contrast, the PEN-treated group with WSLE NE showed marked improvement in behavioral performance and histopathological alteration with a significant increase in antioxidant enzyme activity and anti-inflammatory cytokines compared to the group administered WSLE alone. The PEN-treated group with WSLE NE in turn significantly downregulated the expression levels of GFAP, APP, vimentin, TGF-β1, Smad2 and Bax in brain tissue. In conclusion, WSLE NE markedly enhanced the permeability of plant extract constituents through the blood brain barrier to boost its neuroprotective effect against PEN-induced neurotoxicity.

## Introduction

Pesticides are one-of-a-kind environmental contaminants that introduced to control pests and protect against crop loss (Klaassen 2013). Although Pesticides have proven to be essential tools in agriculture, there are significant concerns over the potentially toxic effects of pesticides on non-target populations, including humans (Bumbăcilă and Putz 2020; Richardson et al. 2019). Pesticides are generally divided into insecticides, herbicides, fungicides, and rodenticides, based on their target pests (Bojarski and Witeska 2020). Fungicides are used ubiquitously in agriculture to control molds (Zubrod et al. 2019). However, many fungicides impair mitochondrial function in non-target populations, which may exacerbate neurodegeneration (Bhagat et al. 2021; Bielská et al. 2021). Triazoles, third-generation fungicides, have low biodegradability; therefore, excessive application could generate many residues in the environment and food products with a potentially deleterious effect on mammalian biological systems, especially the nervous system (Sanchez et al. 2020). PEN is a systemic triazole fungicide with preventive and curative properties for powdery mildew in grapes, pome and stone fruit, cucurbits, and strawberries (Derbalah et al. 2011). It inhibits the fungi development via disrupting sterol synthesis within cell membranes (Percival and Haynes 2008). Recent studies have revealed that exposure to PEN causes neurotoxicity with neuropathological lesions in the brain and peripheral nervous system by inducing oxidative stress in brain tissues (Chaâbane et al. 2017a, 2017b; El-Shershaby et al. 2020a; Jia et al. 2020a; Morgan et al. 2021a). Furthermore, the inhibition of depolarization-evoked calcium influx, which likely reduces dopaminergic neurotransmission, could represent one of the potential mechanisms mediating the neurotoxic impact of PEN (Heusinkveld et al. 2013).

There are numerous reports on herbal products that can treat or delay neurodegenerative diseases (Zahiruddin et al. 2020). *W. somnifera*, known as Ashwagandha or Indian ginseng, has been declared to display various properties, including anti-inflammatory, antioxidant, memory-enhancing, and antiparkinsonian (Gopukumar et al. 2021; Sikandan et al. 2018; Singh et al. 2018). The major bioactive ingredients of WSLE are withanone, withanolide, and withaferin, which are naturally occurring C28 steroids (Singh et al. 2021). Researchers have recently inspected the neuroprotective activity of WSLE by using many neuronal disorder models, and the outcomes are expected to be positive (Birla et al. 2019; Gupta and Kaur 2019; Hosny et al. 2021; Siddiqui et al. 2021; Speers et al. 2021; Wongtrakul et al. 2021). WSLE was hypothesized to be a muscarinic acetylcholine receptor agonist that can regulate memory processes, cognitive function, neuronal excitability, differentiation, dendritic growth, and the synaptic plasticity in the rat hippocampus (Elhadidy et al. 2018; Gautam et al. 2016; Konar et al. 2019). Despite the enormous therapeutic applications of WSLE, the main problem associated with the central nervous system is the inability of its active constituents to cross the blood–brain barrier (Syed et al. 2021a; Vareed et al. 2014). To overcome this obstacle, different formulations of nanomaterials have displayed comprehensive and effective drug delivery for the control of neurological disorders (Chatterjee et al. 2019; Nasr and Wahdan 2019). Inside the nanoparticles, nanoemulsions, kinetically stable biphasic colloidal dispersions exfoliated through amphiphilic surfactants, are promising nanoparticulate systems to overcome the challenges of CNS drug delivery (Chrastina et al. 2018; Karami et al. 2019). Nanoemulsions have several benefits in oral delivery of pharmaceutical compounds, including enormous loading capacity of hydrophobic materials, improved absorption and bioavailability, and enhanced protection against chemical or enzymatic degradation, which may provide a solution to the delivery of difficult-to-formulate phytopharmaceuticals, where stability remains a major concern (Ding et al. 2018; Pathak et al. 2018). Therefore, in the present study, we aimed to prepare a WSLE NE that could enhance the bioavailability of the plant extract in brain tissue and evaluate its efficiency against PEN-triggered neurotoxicity in albino rats.

## Materials and methods

### Chemicals

PEN (purity: 99.18%, CAS No. 66246–88-6, Catalog No: HY-135761, MedChemExpress, USA).

### Plant sample and extraction

*W. somnifera* leaves were collected from field-grown plants in the Faculty of Agriculture Botanical Garden, Mansoura University, Mansoura, Egypt, in October 2022. These plants were identified through the available literature and authenticated by a special botanist. They were stored in the department's herbarium lab with voucher number WS02022. The collected leaves were cleaned and air-dried for 6 days before being ground to a fine.

powder and then subjected to extraction using the Soxhlet method (Nile et al. 2019). Powdered plant samples (30 g) were extracted with 500 mL of 80% methanol at 60 °C in a Soxhlet apparatus for 12 h. The obtained extract was filtered, centrifuged, and concentrated using a rotatory evaporator under reduced pressure at 60 °C, lyophilized, weighed, re-suspended in saline for use as a stock solution, and stored at 4 °C for further studies.

### Characterization of compounds in WSLE Using LC–MS/MS analysis

LC–MS/MS Analysis was performed through UHPLC/QTOF-MS (Agilent, Santa Clara, CA, USA) technique in both positive and negative modes using Exion LC High flow LC system (Sciex hardware, ON, Canada) for chromatographic separation coupled with triple TOF 5600 + IDA acquisition and QTOF-MS/MS detector Mass according to (Hegazy et al. 2021). 50 mg of WSLE was mixed with 1 mL of the mobile phase reconstitution solvent (water: methanol: acetonitrile, 2:1:1). Stock solution was further diluted with the reconstitution solvent (injected concentration, 2.5 µg/µL) then 10 μL of sample was injected for a chromatographic run in positive and negative modes. 10 μL of reconstitution solvent was used as a blank sample. Analysis of peaks was carried out using PeakView^®^ 1.2 Softwares (SCIEX, Framingham, MA, USA). The ReSpect-positive and ReSpect-negative databases (2737 and 1573 records, respectively) were used as reference databases. Predicting the identity of unknown compounds supported by Kyoto Encyclopedia of Genes and Genomes (KEGG) and GNPS spectral libraries (NIST14, MoNA, and Respect).

### Preparation of WSLE NE

The WSLE NE was prepared using an oil-in-water system through a high-energy emulsification process, as previously described (Hussein and El-Naggar 2021; Prashar et al. 2021). To prepare the aqueous phase, 5 mL of Tween 80 was dispersed in 50 mL distilled water and stirred for 15 min at 25 °C. For the oil phase, 5 mL linoleic acid and lecithin (250 mg) were dissolved in 35 mL of *W. somnifera* extract (5%) and stirred for 15 min at 40 °C. Subsequently, the oil phase was added dropwise to the aqueous phase and stirred for 30 min. Finally, the mixture was subjected to ultrasonication using a probe sonicator (Ultrasonics, USA) with a 5-s pulse rate for 5 min in an ice bath. The formulated WSLE NE was stored in a refrigerator at 4 °C until further characterization and analysis.

### Characterization of WSLE NE

#### Droplet size measurement

Dynamic light scattering (DLS) was used to evaluate the droplet size and polydispersity index (PDI) of the prepared nanoemulsion using a Zetasizer Nano ZS (Malvern, UK), and 0.1% (v/v) of the sample was mixed in 0.05 M phosphate buffer (pH 7), and three measurements were taken for the nanoemulsion sample.

#### Morphology of the nanoemulsion

The morphological characteristics of the Ashwagandha nanoemulsions were observed by transmission electron microscope (TEM) analysis. One drop of ten-fold diluted NE sample solution was deposited onto a carbon-coated copper grid and then negatively stained with 2% phosphotungstic acid for 1 min, as previously reported (Li et al. 2016; Mazzarino et al. 2018). All samples were dried overnight at room temperature before capturing the TEM images.

### Experimental animals

Forty adult male Albino Wistar rats (160 ± 10 g) were purchased from the Faculty of Pharmacy animal unit, Mansoura University, were acclimatized for 2 weeks before the experimental study under conventional lightening system and ambient temperature (23 ± 2 °C) with free access to feed and water. All animals were handled and housed in accordance with the formal approval of Mansoura University's ethical committee (MU-ACC (VM.R.23.09.126)).

### Experimental design

Rats were randomly divided into five groups (*n* = 6 per group) and the experimental design was planned as follows: group I, control (received 1 mL DW daily); group II, administered WSLE NE (200 mg/kg body weight) based on (Khalil et al. 2021; Konar et al. 2011); Group III, gavaged 1/20 LD_50_ of PEN (100 mg/kg body weight), which was evaluated according to the up-and-down procedure, data were analyzed through the AOT 425 statistical program according to the Organization for Economic Co-operation and Development (OECD) guidelines (Bruce 1985; OECD 2001); group IV, received simultaneous PEN (100 mg/kg body weight) and WSLE (200 mg/kg body weight); and group V, gavaged PEN (100 mg/kg body weight) and WSLE NE (200 mg/kg body weight). All animals were treated daily by oral gavage for 6 weeks according to Hashem et al. (2023).

### Assessment of behavioral neurotoxicity

#### Open field test

This test was performed to evaluate exploratory, locomotor, and anxiety-related behaviors in rodents (Crépeaux et al. 2017). The experimental animals were placed in a square box (100 × 70 × 40 cm) that divided into 16 equal squares. Each experimental rat was abandoned freely explored the square box for 5 min. Freezing time, rearing frequency, and time invested in the center area were documented (Ghasemnejad-Berenji et al. 2021; Wang et al. 2020).

#### Forced swimming test (FST) for depression activity

The FST was conducted according to Petit-Demouliere et al. (2005). Each rat was placed into transparent cylindrical containers (30 × 45 cm) filled with water for 15 min. The motionless rats made movements only to keep their heads above the water's surface; immobility time was recorded.

#### Y-maze test

Y-maze consisted of three equal-sized arms (50 cm long, 20 cm high, and 15 cm wide), labeled A, B, and C, respectively. Each rat had the opportunity to freely explore the maze at the end of one arm for 8 min with manual recording of the sequence of arm entries. Spontaneous alternation behavior which referred to the entry of all three arms on alternating triplets or successive choices (ABC-BAC) is regarded as a measure of spatial memory (Yamada et al. 1999), the percentage of spontaneous alternation behavior was calculated from the following equation (Ghafouri et al. 2016):

#### Hot plate test

The animals were placed on a hot plate maintained at 52 °C and the latency time (s) between contact with the heated plate and the rat’s response to the heat stimulus including paw-licking and jumping off was recorded. This reaction time was taken as the end point and the cut of time was considered at 20 s to avoid tissue damage (Semis et al. 2021).

## Biochemical and histological examination

### Tissue preparation

After 6 weeks, all animals were anesthetized by thiopental sodium (30 mg/kg) b. wt., injected intraperitoneal according to Barai et al. (2019). Afterwards, rats were sacrificed, and brains were removed quickly from the skull and rinsed several times in cold normal saline. Finally, brain tissues were divided into three parts for studying biochemical, molecular and histopathological changes. The sciatic nerve was surgically dissected from the right lateral femoral region for histopathological examination.

For the preparation of biochemical analysis, brain samples were minced and homogenized (10% w/v) in ice-cold phosphate buffer saline (0.01 M, pH 7.4), then centrifuged at 12,000 Xg at 4 °C for 20 min, and the resultant supernatant was stored at – 80 °C until used (Posadas et al. 2009).

### Assessment of inflammatory cytokines

The concentrations of inflammatory cytokines were evaluated in brain tissues using an ELISA kit for interleukin 6 (IL-6) (BMS625), interleukin 10 (IL-10) (BMS629), and tumor necrosis factor-α (TNF-α) (BMS622), (Invitrogen, MI, USA). Tissue supernatants were removed, and cytokine concentrations were measured through a microplate reader (Molecular Devices, CA, USA) at 450 nm according to the manufacturer’s guidelines and results were expressed as pg/mL (Jiang et al. 2021).

### Assessment of acetylcholinesterase (AChE) activity

AChE activity expressed as (nmol/min/mg tissue) was measured in the brain tissue based on Ellman’s method (Madiha et al. 2021). Briefly, 0.4 mL of brain homogenate (20%), 100 μL 5,5′-dithio-bis-nitrobenzoic acid (DTNB), and 2.6 mL phosphate buffer (pH 8.0) were mixed then the reaction started by adding 5 μL acetylthiocholine iodide, used as a substrate, and the absorbance was detected at 412 nm.

## Estimation of oxidative stress and antioxidant enzymes activity

### Measurement of MDA level

MDA was measured by the double heating method (Odukoya et al. 1994). In short, 2.5 mL of TCA (15%) was added to 0.5 mL of homogenized sample of brain tissue then placed in a boiling water bath for 15 min and centrifuged after cooling at 3000 g for 10 min. Equal amounts of supernatant and TBA solution (0.67%) were mixed and placed in a boiling water bath (95 °C) for 20 min, the pink-colored supernatant was measured at 532 nm against the Blank solution and Tissue MDA levels were expressed as nmol/g tissue (Haider et al. 2014).

### Assessment of reduced GSH content

The GSH level was measured with respect to Moron et al. (1979), where equal amounts of brain tissue homogenate (10%) trichloroacetic acid (10%) were mixed and centrifuged for 5 min at 3000 × *g* then supernatant collected since 1 mL of supernatant added to 0.1 mL DTNB (0.04%), the yellow-colored TNB was measured at wavelength of 412 nm. GSH level was quantified as nmol/g tissue (Mohammadzadeh et al. 2018).

### Determination of superoxide dismutase (SOD) activity

Brain SOD activity was estimated based on the Marklund method (Marklund and Marklund 1974). Brain tissue was homogenized in Tris–HCl buffer (0.1 M, pH 7.4), then centrifuged at 12,000 × g for 15 min and 100 μL of supernatant was added to 0.05 M Tris–HCl buffer, 2.64 mM Pyrogallol and 1 mM EDTA and the absorbance recorded at 420 nm for 5 min. The SOD activity was quantified as U/g tissue (Muthulakshmi et al. 2018).

### Measurement of catalase (CAT) activity

The catalase activity was quantified on the basis of Aebi et al. (1972). In brief, the reaction mixture was composed of 1 mL of phosphate buffer (0.05 M), 0.4 mL of 0.03 M hydrogen peroxide, and 0.1 mL of tissue homogenate (10%); the decomposition rate of H_2_O_2_ was followed at 240 nm for 60 s. at room temperature. CAT activity was quantified as units per g tissue (Afolabi et al. 2019).

### Assessment of glutathione S-transferase (GST) activity

GST activity was measured through the assay of Habig et al. (1974) by using 4-chloro,1–3-dinitrobenzene (CDNB) as a substrate. 20 μL tissue supernatant was added to 150 μL of potassium phosphate buffer (0.1 M), 20 μL of glutathione (200 mM), and 10 μL of CDNB (100 mM) to start the reaction. The activity of GST was determined spectrophotometrically at 340 nm every 30 s for 5 min and calculated as U/g tissue.

### RT-PCR analysis

#### RNA extraction

Total RNA was extracted from 50 mg of the second part of the brain tissues using the RNeasy Mini Kit (iNtRON Biotechnology, Inc., South Korea) following the guidelines of the manufacturer procedures. The quality of the isolated RNA was qualified by 1.5% agarose gel electrophoresis. Meanwhile, the concentration of the obtained RNA was checked using Nanodrop (Thermo Science, USA). The extracted RNA was subsequently reverse transcribed by a QuantiTect Reverse Transcription kit (Qiagen, Heidelberg, Germany) according to the manufacturer’s guidelines. Then, cDNA samples were kept at − 20 °C until further use.

### QRT-PCR analysis

Specific primer sequences of *APP*,* GFAP,* vimentin*, TGF-β1* and *Bax* genes used in RT-qPCR were tabulated in (Table 1). In brief, the qPCR investigation was estimated in a Rotor-Gene Q apparatus with a QuantiTect® SYBR® Green PCR kit (SensiFast™ SYBR Lo-Rox kit, Bioline) under the following conditions: 95 °C for 10 min, then 40 cycles of 95 °C for 15 s, 60 °C for 15 s, and 72 °C for 15 s. After that, the melt-curve analysis was done to confirm the specificity of the qPCR. The necessary relative fold change of the target genes expression profile was calculated using the comparative 2 ^−∆∆Ct^ method with *β*-actin as a housekeeping gene (internal control) for standardization of the target genes expression levels (Livak and Schmittgen 2001).

**Table 1:** Sequences of primers used for RT-qPCR analysis:

Target gene	Primers sequences	s Reference	PCR product size
*APP*	F: 5`-TGGGTTGACAAACATCAAGACAGAA-′3	Ying-Cai et al. (2007**)**	135 bp
	R: 5`-GCACCTTTGTTTGAACCCACATC -′3		
*GFAP*	F: 5`-CAGACTTTCTCCAACCTCCAG -′3	Doorn et al. (2015**)**	138 bp
	R: 5`-CTCCTGCTTCGAGTCCTTAATG-′3		
*Vimentin*	F: 5`-TGTCCAAATCGATGTGGATGTTTC-′3	Rogel et al. (2011**)**	117 bp
	R: 5`-TTGTACCATTCTTCTGCCTCCTG-′3		
*TGF-β1*	F: 5`-CCTGCAAGACCATCGACATG-′3	Saad et al. (2019**)**	85 bp
	R: 5`-GCGAGCCTTAGTTTGGACAG-′3		
*smad2*	F: 5`-AGCAGGAATTGAGCCACAGAGT-′3	Wang et al. (2021a**)**	175 bp
	R: 5`-TGGCTGCAAATCCAAGCTGT-′3		
Bax	F: 5`-TGCTTCAGGGTTTCATCCA-′3	Samarghandian et al. (2022**)**	111 bp
	R: 5`-GACACTCGCTCAGCTTCTTG-′3		
*Β-actin*	5'-TCCTCCTGAGCGCAAGTACTCT-′3	Banni et al. (2010**)**	153 bp
	5'-GCTCAGTAACAGTCCGCCTAGAA-′3		

## Histopathological and immunohistochemical evaluation

The third part of the brain tissue and peripheral sciatic nerve were fixed in 10% neutral formalin, dehydrated, and embedded in paraffin based on Bancroft and Layton (2012) technique, then sliced into 5 µm thick sections. The coronal brain cerebral cortex sections were stained with crystal violet. Transverse sciatic nerve sections were stained with H and E, then observed by light microscope. Histomorphometry analysis of the sciatic nerve sections, the percentage area of the nerve fiber was measured in five different areas from each section.

Assessment of tau protein and NF-κB immunohistochemical expression in cerebral cortex and sciatic nerve, respectively, was carried out following the same method of Abomosallam et al. (2023). Five random fields from each section were taken from the original micrographs through a digital camera with an original magnification of ×100 (Nikon Eclipse E200-LED, Tokyo, Japan). The histomorphology of the sciatic nerve and the percentage of immunohistochemical expression were assessed via ImageJ software.

## Statistical analysis

All data values are presented as mean ± SD and considered statistically significant at *P*-value ≤ 0.01. Data were analyzed by one-way ANOVA and Tukey's post hoc test via SPSS version 20.0 (SPSS, Inc., Chicago, IL, USA) and GraphPad Prism 5. *P*-value ≤ 0.01 considered as statistically significant.

## Results

### LC–MS/MS analysis of WSLE

A total of 91 compounds of different chemo-types were detected and identified in WSLE in both ionization modes (Supplementary) comprising 5 withanolides, 21 flavonoids, 11 phenolic acids, ten organic acids, 13 amino acids, 4 fatty acids, 3 coumarins, one anthocyanidin, one auren and others (Table 2).

**Table 2:** LC–MS/MS analysis of WSLE

No.	Title	RT(min)	[M+H]^+^	[M-H]^-^	Formula	Err.	MS/MS Positive	MS/MS Negative
1	6,7-epoxy-3,5,20-trihydroxy-1-oxowitha-24-enolide	11.063	506.3111^c^		C_28_H_40_O_7_	− 0.3	489, 471, 453, 435, 417, 399, 381, 303, 299, 281, 263, 171, 169, 125	
2	Withaferin A	12.431	488.3007^c^		C_28_H_38_O_6_	0.0	471, 453, 435, 417, 399, 283, 265,175,171, 95, 67	
3	12-deoxy withstramonolide	13.120	488.3008^c^		C_28_H_38_O_6_	0.4	453,435, 399, 299, 281, 263, 95 and 67	
4	Withanolide A	14.977	941.5414^d^		C_28_H_38_O_6_	0.5	923, 905, 887, 869, 471, 453, 435, 417, 399, 285, 267, 263,175, 171, 169, 123	
5	Withanone	15.001	941.5434^d^		C_28_H_38_O_6_	2.6	923, 905, 887, 869, 471, 453, 435, 417, 399, 285, 267, 265, 169, 123	
6	Malic acid	0.991		133.0137	C_4_H_6_O_5_	1.5		115, 89, 73, 71
7	Mucic acid	1.004		209.0290	C_6_H_10_O_8_	− 0.8		129, 85, 75, 73, 71, 59, 57
8	Maleic acid	1.042		115.0035	C_4_H_4_O_4_	0.8		71
9	(-)-Shikimic acid	1.080		173.0482	C_7_H_10_O_5_	− 0.7		155, 137, 129 111, 93, 73
10	Citramalate	1.088		147.029	C_5_H_8_O_5_	2.2		129, 87, 85, 69
11	Hydroxylysine	1.114		161.0452	C_6_H_14_N_2_O_3_	− 0.8		143, 115
12	Lactic acid	1.117		89. 0232	C_3_H_6_O_3_	− 0.5		89
13	Thymidine-monophosphate	1.122		321.0835	C_10_H_15_N_2_O_8_P	− 3.8		195, 176, 125, 97, 78
14	2-Aminoadipic acid	1.130		160.0620	C_6_H_11_NO_4_	0.3		142, 116, 114, 98
15	L-Glutamic acid	1.144		146.0457	C_5_H_9_NO_4_	6.3		128, 102, 84, 71
16	Quinic acid*	1.146		191.0550	C_7_H_12_O_6_	0.2		173, 127, 93, 85
17	Glyceric acid	1.178		105.0210	C_3_H_6_O_4_	− 2.2		75, 73
18	Histidine	1.193		154.0616	C_6_H_9_N_3_O_2_	3.6		137, 108, 93, 81, 66
19	Glycine-Betaine	1.217	118.0860		C_5_H_11_NO_2_	0.3	59, 58	
20	Cytidine	1.221		242.0771		0.4		152, 109, 81
21	Dihydroxybenzoic acid	1.245		153.0172	C_7_H_6_O_4_	0.8		109, 108,91
22	Choline	1.271	104.1064		C_5_H_14_NO	− 0.5	104, 60, 58	
23	Sucrose	1.273		341.1003	C_12_H_22_O_11_	0.1		179, 161, 149, 89
24	DL-alpha,epsilon-Diaminopimelic acid	1.279		189.0867	C_7_H_14_N_2_O_4_	0.6		189, 171, 128,127, 111
25	L-Asparagine	1.318	133.0604		C_4_H_8_N_2_O_3_	− 0.4	116, 88, 87, 74, 70	
26	Cytosine	1.370	112.0501		C_4_H_5_N_3_O	0.1	95, 94, 69	
27	*p*-Coumaric acid*	1.384		163.0413	C_9_H_8_O_3_	− 0.9		119, 93
28	Proline	1.406	116.0335		C_5_H_9_NO_2_	0.3	70	
29	Citraconic acid	1.448		129.0182	C_5_H_6_O_4_	0.3		85
30	Tyrosine	1.452	182.0823	180.0660	C_9_H_11_NO_3_	2.5	165, 147, 136, 123, 91	163, 119, 93, 72
31	L-5-Oxoproline	1.579	130.0497		C_5_H_7_NO_3_	− 1.0	84, 56	
32	D-Alloisoleucine	1.683	132.1018		C_6_H_13_NO_2_	− 2.8	86, 69	
33	Uridine	1.707		243.0623	C_9_H_12_N_2_O_6_	− 0.9		200, 152, 110, 82
34	Guanosine	1.708		282.0852	C_10_H_13_N_5_O_5_	0.4		150, 133, 108, 80,78
35	Piperidine	1.859	86.0622		C_5_H_11_N	0.5	69, 56	
36	Thymidine	1.946		241.0815	C_10_H_14_N_2_O_5_	− 1.8		197, 151, 125
37	Trans-Cinnamic acid	2.059		147.0452	C_9_H_8_O_2_	0.0		119, 103, 93, 77, 68
38	Phenylalanine	2.084		164.0705	C_9_H_10_NO_2_	− 0.3		147, 103, 91,72
39	Caffeic Acid	2.094		179.0344	C_9_H_8_O_4_	0.7		135, 134
40	Caffeoylquinic acid	2.111		353.0859	C_16_H_18_O_9_	− 2.4		191, 179, 173, 135
41	*P*-Nitrophenol	2.285		138.0187	C_6_H_5_NO_3_	1.1		108
42	Adenine	2.310	136.0618		C_5_H_5_N_5_	0.1	119	
43	Pantothenic acid (Vitamin B_5_)	2.350	220.1183		C_9_H_17_NO_5_	1.7	202, 184, 90	
44	L-Tryptophane	3.119	205.0973		C_11_H_12_N_2_O_2_	− 0.9	188, 170, 159, 146, 118	
45	Adenine	3.167		134.0462	C_5_H_5_N_5_	− 1.0		107, 92, 65
46	7-hydroxy-4-methylcoumarin	3.865	177.0546		C_10_H_8_O_3_	− 0.3	149, 135, 121, 117, 89	
47	3-Formylindole	3.926	146.0598		C_9_H_7_NO	− 1.8	118, 91	
48	*P*- hydroxybenzoic acid	3.938		137.0236	C_7_H_6_O_3_	1.8		93, 65
49	Salicylic acid	4.039		137.0247	C_7_H_6_O_3_	− 0.3		93, 65
50	Quercetin3-*O*-rutinoside-7-*O*-glucoside	4.220	773.2127		C_33_H_40_O_21_	− 1.0	627, 465, 303	
51	Quercetin 3-*O*-robinoobiside-7-*O*- glucoside	4.235	773.2115		C_33_H_40_O_21_	− 2.6	627, 465, 303	
52	Quercetin-*O*-hexosyl-*O*-tetraacetyl hexoside	4.245	795.1973		C_35_H_38_O_21_	− 0.7	633, 465, 303	New
53	Apigenin-*O*-pentoside-*O*-hexoside	4.248		609.1892^a^	C_26_H_28_O_14_	− 0.8		563, 401, 269
54	Quercetin	4.317	303.0470		C_15_H_10_O_7_	5.4	286	
55	Hydroxymethylcoumarin-*O*-hexoside	4.449	339.1072		C_16_H_18_O_8_	0.8	177, 147, 134	
56	6,7- dihydroxycoumarin	4.684	179.0336	177.0182	C_9_H_6_O_4_	− 0.4	151, 133, 123, 105, 77	149, 133, 121, 105
57	kaempferol-3-*O*-rutinoside-7-*O*-glucoside	4.746	757.2207		C_33_H_40_O_20_	2.8	611, 449, 287	
58	kaempferol-3-*O*- robinoobiside -7-*O*-glucoside	4.770	757.2208		C_33_H_40_O_20_	3.0	611, 449, 287	
59	Dicaffeoylquinic acid	5.132		515.1235	C_25_H_24_O_12_	9.8		353, 335, 191, 179, 173,161,134
60	(-)-Riboflavin	5.167	377.1466		C_17_H_20_N_4_O_6_	2.8	243, 198, 172	
61	Phenylacetic acid	6.342		135.0449	C_8_H_8_O_2_	− 6.2		93, 92
62	Quercetin 3-*O*-glucoside	6.346		463.0862	C_21_H_20_O_12_	− 1.8		301, 300, 271, 255, 151
63	Delphinidin-3-*O*-(6''-*O*-alpha-rhamnopyranosyl-beta-glucopyranoside)	6.382	611.1599^b^		C_27_H_31_O_16_	− 0.9	465, 303	
64	Rutin	6.424	611.1616	609.1430	C_27_H_30_O_16_	1.6	303	301, 300, 299
65	Vanillic acid	6.485		167.0337	C_8_H_8_O_4_	− 0.9		152, 108
66	Maritimetin-6-*O*-glucoside	6.604		447.0924	C_21_H_20_O_11_	0.5		285, 284, 151, 135
67	Kaempferol3-*O*-rutinoside	6.642		593.1496	C_27_H_30_O_15_	− 0.9		285
68	Isorhamnetin-3-*O*-rutinoside	6.683		623.1632	C_28_H_32_O_16_	1.0		577, 315, 314
69	Quercetin 3-*O*-galactside	6.700	465.1029		C_21_H_20_O_12_	0.4	303	
70	Kaempferol-3-*O*-(coumaroyl)-glucoside	6.720		593.1483	C_27_H_30_O_15_	− 2.9		447, 285, 284
71	Quercetin-*O*-rhamnosyl-pentoside	6.744	581.2955	579.1372	C_26_H_28_O_15_	− 0.3	435, 303	301, 300
72	Indole-3- acetic acid	6.792		174.0563	C_10_H_9_NO_2_	2.1		130,128
73	Quercetin3-*O*-rhamnoside	6.902		447.0926	C_21_H_20_O_11_	− 0.5		301, 300, 271, 255
77	Luteolin	7.037	287.0539		C_15_H_10_O_6_	− 0.9	153, 131	
78	Kaempferol	7.107	287.0533		C_15_H_10_O_6_	− 4.3	287, 184, 153	
79	Apigenin-feruloyl-pentoside	7.111		577.1912	C_28_H_34_O_13_	− 0.6		401, 269, 193, 175,160, 134
80	Luteolin-7-*O*-rutinoside	7.082	595.1629		C_27_H_30_O_15_	0.4	449, 287	
81	Kaempferol -7-*O*-neohesperidoside	7.148	595.1657		C_27_H_30_O_15_	− 0.1	449, 287	
82	Isorhamnetin-*O*-neohesprioside	7.202	625.1777		C_28_H_32_O_16_	2.1	479, 317	
83	Naringenin-*O*-glucosyl rhamnoside	7.357		579.2027	C_27_H_32_H_14_			417,271, 193, 181, 179
84	Ferulic acid	8.430		193.0511	C_10_H_10_O_4_	1.1		178, 161, 133
85	Naringenin	9.775		271.0619	C_15_H_12_O_5_	− 1.8		177, 151, 119
86	Syringic acid	12.499		197.1588	C_9_H_10_O_5_	0.4		167, 153
87	Nicotinamide	15.123	123.0804		C_6_H_6_N_2_O	− 1.5	108, 81, 55	
88	Linolenic acid	19.571		277.2175	C_18_H_30_O_2_	0.2		337, 261, 259, 233, 205,179
89	Linoleic acid	21.801		279.2331	C_18_H_32_O_2_	4.4		279
90	Palmitic acid	23.205		255.2322	C_16_H_32_O_2_	− 0.5		255
91	Oleic acid	23.800		281.2472	C_18_H_34_O_2_	− 1.1		281

a [M-H + FA]-; b [M] + c [M + NH4] + ; d [2M + H] +

WSLE contains steroidal lactones, commonly known as withanolides and have been detected in both ionization modes, especially in the positive ion mode through their fragmentation pattern. Consequently, five bioactive withanolides have been tentatively identified as 6,7-epoxy-3,5,20-trihydroxy-1-oxowitha-24-enolide with molecular ion peak at *m/z* 506.3111 [M+NH_4_]^+^ (calc. C_28_H_44_O_7_N^+^), *R*_t_ 11.063 min with error − 0.3 and showed peaks at *m/z* 489, 471, 453, 435, 417, 399, 381, 303, 299, 281, 263, 171, 169 and 125 confirmed the presence of C-5 OH, an epoxy group at C-6/C-7, C-20 OH and absence of C-27 OH and C-17 OH while peak at *m/z* 125 corresponding to the fission of C-20/C-22 for *α*-*β* unsaturated *δ*-lactone; withaferin A with molecular ion peak at *m/z* 488.3007 [M+NH_4_]^+^ (calc. C_28_H_42_O_6_N^+^), *R*_t_ 12.431 min with error 0.0; 12-deoxy withastramonolide demonstrated molecular ion peak at *m/z* 488.3007 [M + NH_4_]^+^ (calc. C_28_H_42_O_6_N^+^), *R*_t_ 13.120 min with error 0.4 and showed peaks at *m/z* 453,435, 399, 299, 281, 263, 95 and 67 confirmed the presence of C-5 OH, an epoxy group at C-6/C-7 and C-27 OH; withanolide A showed molecular ion peak at *m/z* 941.5414 [2M+H]^+^ (calc. C_56_H_77_O_12_^+^) due to the dimerization of the molecular ion at *R*_t_ 14.977 min with error 0.5 and gave peaks at *m/z* 923, 905, 887, 869, 471, 453, 435, 417, 399, 285, 267, 263, 169 and 123 proven the presence of C-5 OH, an epoxy group at C-6/C-7, C-20 OH and the absence of C-27 OH; and withanone (5) with molecular ion peak at *m/z* 941.5434 [2M+H]^+^ (calc. C_56_H_77_O_12_^+^), *R*_t_ 15.001 min with error 2.6 (Table 2) and supplementary Fig. S3-S7).

Fragmentation patterns of most flavonoids displayed apparent [M-H]^−^ or [M + H]^+^ ions due to the presence of hydroxyl, phenolic and methyl as subgroups linked to the flavonoid aglycone. The sugars moieties linked to the flavonoid aglycone could be identified by the presence of the fragment ions of their corresponding aglycones. In the rutinoside moiety (1 → 6), the fragment [M + H-308]^+^ is the base peak ion, while in neohesperidoside moiety (1 → 2), the base peak ion is [M + H-rhamnosyl]^+^. Flavone aglycone (luteolin 69) and its diglycosides (luteolin 7-*O*- rutinoside 72) were identified with their characteristic ion peaks at *m/z* 287.0539 [M + H]^+^ and 595.1629 [M + H]^+^; while (apigenin-*O*-pentoside-*O*-hexoside 48) and (apigenin-feruloyl-pentoside 71) were detected at *m/z* 609.1892 [M-H + FA]^−^ and 577.1912 [M-H]^−^. Flavonol subclass, kaempferol and kaempferol glycosides were noticed displaying the characteristic fragment ions at *m/z* 287 [M + H]^+^, 285 [M-H]^−^ and 284 [M-H–H]^−^. The characteristic parent ion peak for kaempferol 70 appeared at *m/z* 287.0533 [M + H]^+^, while that of kaempferol diglycosides appeared at *m/z* 593.1496 [M + H]^+^ and *m/z* 595.1657 [M + H]^+^, corresponding to kaempferol 3-*O*-rutinoside 62 and kaempferol 7-*O*-neohesperidoside 73. Kaempferol triglycosides were tentatively identified as kaempferol-3-*O*-rutinoside-7-*O*-glucoside (52; *m/z* 757.2207 [M + H]^+^) and kaempferol-3-*O*-robinobioside-7-*O*-glucoside (53; *m/z* 757.2208 [M + H]^+^). Quercetin and its derivatives were also recognized with characteristic fragment ions at *m/z* 301 [M-H]^−^ and 303 [M + H]^+^. Quercetin (49; *m/z* 303.0470 [M + H]^+^) and its derivatives were annotated as quercetin-3-*O*-rutinoside-7-*O*-glucoside (45; *m/z* 773.2127 [M + H]^+^) and quercetin-3-*O*-robinobioside-7-*O*-glucoside (46; *m/z* 773.2115 [M + H]^+^). Other derivatives were annotated as rutin (59; *m/z* 611.1616 [M + H]^+^), quercetin-*O*-rhamnosyl-pentoside (66; *m/z* 581.2955 [M + H]^+^, 579.1372 [M-H]^−^) which confirmed via its ion fragmentations in both modes (Table). Quercetin-3-*O*-glucoside (57; *m/z* 463.0862 [M-H]^−^), quercetin-3-*O*-galactoside (64; *m/z* 465.1029 [M + H]^+^) and quercetin 3-*O*-rhamnoside (68; *m/z* 447.0926 [M-H]^−^) were also detected. quercetin-*O*-hexosyl-*O*-tetraacetyl hexoside (47; *m/z* 795.1973 [M + H]^+^) was tentatively identified for the first time from nature based on its fragmentation pattern which revealed ions at *m/z* 633 [M + H-hexosyl]^+^, *m/z* 465 [M + H-hexosyl-168 (2 × 42)]^+^ and *m/z* 303 [M + H-hexosyl-168–162]^+^ for the successive loss of an *O*-hexosyl moiety, four acetyl groups, and an additional *O*-hexoside moiety (SI. Figs  3, 4). Moreover, diglycosides of isorhamnetin were appointed as isorhamnetin-3-*O*-rutinoside (63; *m/z* 623.1632 [M-H]^−^) and isorhamnetin-*O*-neohesperidoside (74; *m/z* 625.1777 [M + H]^+^). Furthermore, two flavanone were detected and tentatively identified as naringenin 77 and naringenin-*O*-glucosyl rhamnoside 75 based on their ion fragmentations with ion peaks at *m/z* 271.0619 [M-H]^−^ and 579.2027 [M-H]^−^.

Anthocyanidin derivative delphinidin-3-*O*-(6''-*O*-alpha-rhamnopyranosyl-beta-glucopyranoside) 58 was tentatively identified with ion peak at 611.1599 [M]^+^, while an aurone (61; maritimetin-6-*O*-glucoside) is detected at *m/z* 447.0924 [M-H]^−^. For coumarins, three compounds were displayed including 6,7-dihydroxycoumarin (51; *m/z* 179.0336 [M + H]^+^ and 177.0182 [M-H]^−^), 7-hydroxy-4-methylcoumarin (41; *m/z* 177.0546 [M + H]^+^) and hydroxymethylcoumarin-*O*-hexoside (50; *m/z* 339.1072 [M + H]^+^).

11 phenolic acids belong to different sub classes such as hydroxybenzoic, hydroxycinnamic, and chlorogenic acids were tentatively identified in *W. somnifera*. The hydroxybenzoic acids derivatives as dihydroxybenzoic acid 16 with precursor ion at *m/z* 153.0172 [M-H]^−^, and a diagnostic fragment 109 [M-H-CO_2_]^−^, *P*-hydroxybenzoic acid 43 with ions at *m/z* 137.0236 [M-H]^−^, 93 [M-H-CO_2_]^−^, salicylic acid 44 with ions at 137.0247 [M-H]^−^, 93 [M-H-CO_2_]^−^, 65 [M-H–CO]^−^, syringic acid (78; *m/z* 197.1588 [M-H]^−^), and vanillic acid (60; *m/z* 167.0337 [M-H]^−^). Additionally, the hydroxycinnamic acids derivatives were attributed as caffeic acid 34 with its characteristic ions at *m/z* 179.0344 [M-H]^−^, 135 [M-H-COOH]^−^, *p*-coumaric acid 22 with peaks at *m/z* 163.0413 [M-H]^−^, 119 [M-H-COOH]^−^, and ferulic acid (76; *m/z* 193.0511 [M-H]^−^). chlorogenic acids members, and dicaffeoylquinic acid (54; *m/z* 515.1235 [M-H]^−^) which displayed base peak at 353 [M-H-162]^−^ and fragments similar to those of caffeoylquinic acid.

Other secondary metabolites were identified as an alkaloid (choline 17) which annotated at *m/z* 104.1064 [M-H]^−^ and the characteristic fragment ion at *m/z* 60 due to the loss of trimethylamine (C_3_H_9_N)^+^; indole-3-acetic acid 67 with ion peaks at 174.0563 [M-H]^−^ and 130 [M-H-CO_2_]^−^; 3-formylindole 42 with ion peaks at 146.0598 [M + H]^+^ and 118 [M + H–CO]^+^; Non-reducing disaccharide; sucrose 18 was also identified through its base peak *m/z* 341.1003 [M-H]^−^ and *P*-nitrophenol 36 with an ion peak at 138.0187 [M-H]^−^.

WSLE also contains steroidal lactones, commonly known as withanolides and have been detected in both ionization modes, especially in the positive ion mode, through their fragmentation pattern. Consequently, three withanolides have been tentatively identified as 12-deoxy withastramonolide; demonstrated molecular ion peak at *m/z* 488 [M + NH_4_]^+^ (calc. C_28_H_42_O_6_N^+^), *R*_t_ 13.12 min with error 0.3 and showed peaks at m/z 453,435, 399, 299, 281, 263, 95 and 67 confirmed the presence of C-5 OH, an epoxy group at C-6/C-7 and C-27 OH, withanolide A; showed molecular ion peak at m/z 941 [2M + H]^+^ (calc. C_56_H_77_O_12_^+^) due to the dimerization of the molecular ion at *R*_t_ 14.98 min with error 0.5 and gave peaks at *m/z* 923, 905, 887, 869, 471, 453, 435, 417, 399, 285, 267, 263, 169 and 123 proven the presence of C-5 OH, an epoxy group at C-6/C-7, C-20 OH and the absence of C-27 OH, and 6,7-epoxy-3,5,20-trihydroxy-1-oxowitha-24-enolide; with molecular ion peak at m/z 506 [M + NH_4_]^+^ (calc. C_28_H_44_O_7_N^+^), *R*_t_ 11.06 min with error -0.3 and showed peaks at *m/z* 489, 471, 453, 435, 417, 399, 381, 303, 299, 281, 263, 171, 169 and 125 confirmed the presence of C-5 OH, an epoxy group at C-6/C-7, C-20 OH and absence of C-27 OH and C-17 OH while peak at *m/z* 125 corresponding to the fission of C-20/C-22 for *α*-*β* unsaturated *δ*-lactone.

Other metabolites, many organic acids were noticed only in the negative mode including malic acid 1, mucic acid 2, maleic acid 3, (−)-shikimic acid 4, citramalate 5, hydroxylysine 6, lactic acid 7, quinic acid 11, glyceric acid 12, citraconic acid 24, phenylacetic acid 56 and trans-cinnamic acid 32 as illustrated in (Table 2). While the observed amino acids were hydroxylysine 6, 2-aminoadipic acid 9, l-glutamic acid 10, histidine 13, glycine-betaine 14, dl-alpha,epsilon-diaminopimelic acid 19, l-asparagine 20, proline 23, tyrosine 25, l-5-oxoproline 26, d-alloisoleucine 27, phenylalanine 33 and l-tryptophane 39.

A pyrimidine nucleobase; cytosine (21; *m/z* 112.0501 [M + H]^+^) was annotated with its respective nucleoside; cytidine (15; *m/z* 242.0771 [M-H]^−^), as well as uridine 28 and thymidine 31 nucleosides were detected at *m/z* 243.0623 [M-H]^−^ and *m/z* 241.0815 [M-H]^−^. Nucleotide, thymidine-monophosphate (8; *m/z* 321.0835 [M-H]^−^) fragmented to yield *m/z* 195 (M-H-126) due to the successive loss of thymine nucleobase, 176 [M-H-126-H_2_O], 125 (C_5_H_5_N_2_O_2_) and 97 (H_2_O_4_P). purine nucleoside; guanosine (29; *m/z* 282.0852 [M-H]^−^) and purine nucleobase; adenine (40; *m/z* 134.0462 [M-H]^−^) were also detected. Additionally, four fatty acids were identified in the negative ion mode with their characteristic peaks including linolenic acid (80; *m/z* 277.2175 [M-H]^−^), linoleic acid (81; *m/z* 279.2331 [M-H]^−^), palmitic acid (82; *m/z* 255.2322 [M-H]^−^) and oleic acid (83; *m/z* 281.2472 [M-H]^−^).

### Characterization of WSLE nanoemulsion

**Fig. 1:**
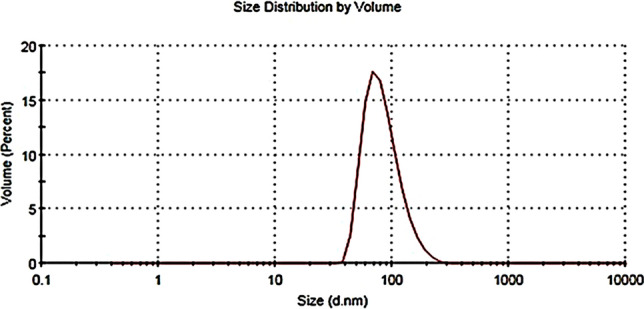
Particle size (nm) distribution of WSLE NE

**Fig. 2:**
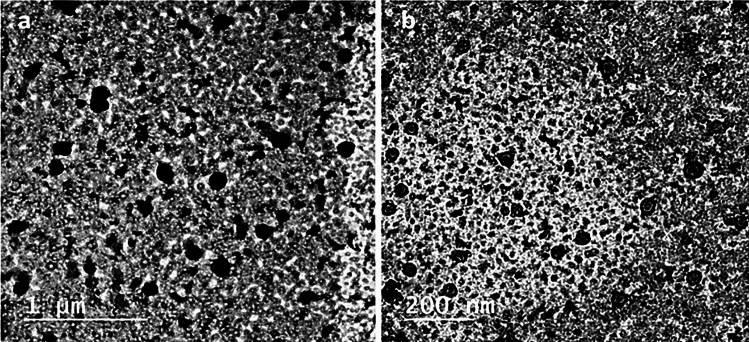
Transmission electron microscope (TEM) micrographs of WSLE NE (1 µm and 200 nm)

The average droplet size of the prepared nanoemulsion was about 86 ± 4.8 nm with a PDI of 0.11 ± 0.02, revealing a narrow size distribution of the nanoemulsion (Fig. 1). The morphological features and accurate size of droplets were observed under TEM. TEM images (Fig. 2) revealed that droplet size was 40–50 nm with uniform spherical shape.

### Assessment of behavioral neurotoxicity

#### Open field test (OFT)

**Fig. 3:**
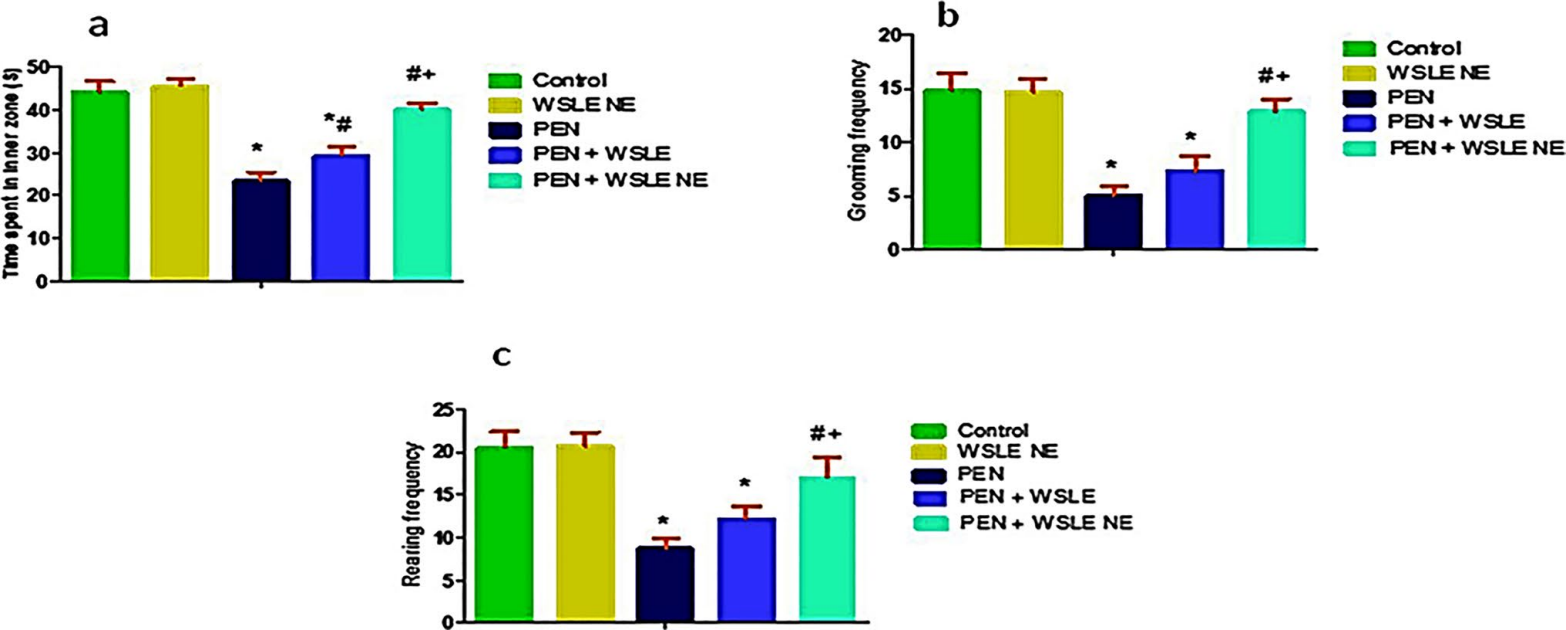
WSLE NE depressed anxiety-related behaviors induced by PEN in rats through enhancing **a** time spent in central squares (s), **b** grooming frequency and **c** rearing frequency in OFT. Values are displayed as mean ± SD (*n* = 6). Data was analyzed by one-way ANOVA and Tukey post hoc test. *, #, + represent significant values when compared to control, PEN-treated and WSLE-treated groups, respectively at *P* < 0.01

OFT test was conducted to assess the exploratory behaviors in rats (Fig. 3). PEN treatment induced notable impacts on the duration spent in the center squares [*F* (4, 25) = 139.88, *P* ≤ 0.01], grooming frequency [F (4, 25) = 75.24, *p* ≤ 0.01] and rearing frequency [*F* (4, 25) = 54.21, *P* ≤ 0.01]. PEN-treated rats showed a significant decrease in time spent in the central squares (*P* < 0.01), grooming frequency (*p* < 0.01), and rearing frequency (*P* < 0.01) in comparison to the control group. However, compared to the PEN-treated rats, WSLE NE-treated animals displayed a relative increase in the duration spent the central area, grooming, and rearing frequency (*P* ≤ 0.01). Furthermore, treatment with WSLE NE had more improving effects (*P* < 0.01) on the alleviation of anxiety-related behaviors than WSLE alone.

### Forced swimming test (FST) for depression activity

**Fig. 4:**
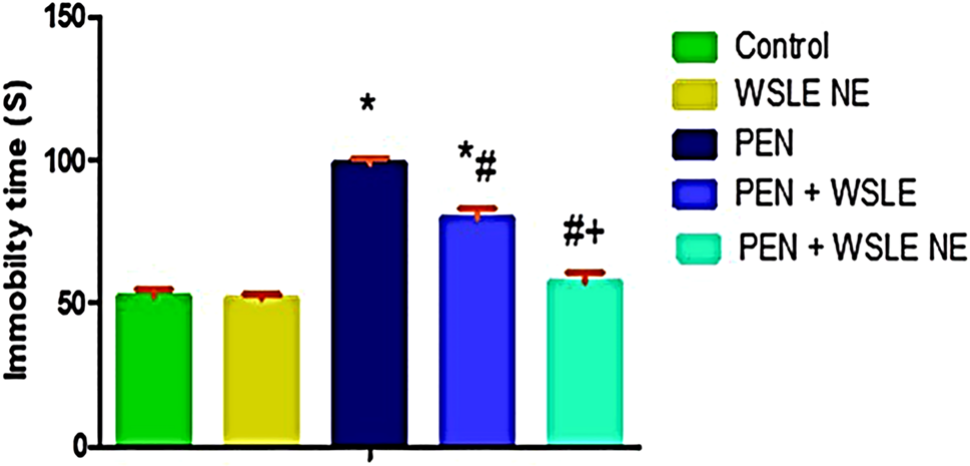
WSLE NE alleviated depression and behavioral despair induced by PEN in rats through significantly decreasing the immobility time (S) in forced swimming test. Data displayed as mean ± SD (*n* = 6) and analyzed by one-way ANOVA and Tukey post-hoc test. *, #, + represent significant values when compared to control, PEN-treated and WSLE-treated groups, respectively at* P* ≤ 0.01

FST was used to evaluate depressive-like behavior and behavioral despair. The data of the FST exhibited that the freezing time was significantly affected following PEN treatment [*F* (4, 25) = 371.938, *P* < 0.01]. PEN treatment showed a significant increase in the immobility time (*P* < 0.01), indicating behavioral despair associated with swim stress (Fig. 4). Tukey’s post-hoc test revealed that the immobility time significantly increased following PEN exposure in comparison to the control animals (*P* < 0.01). On the other hand, WSLE NE-treated animals showed a notable decrease in the immobility time compared to PEN-treated rats (*P* < 0.01). A more significant reduction in immobility time was observed following treatment with WSLE NE in comparison to WSLE.

### Y-maze test

**Fig.5:**
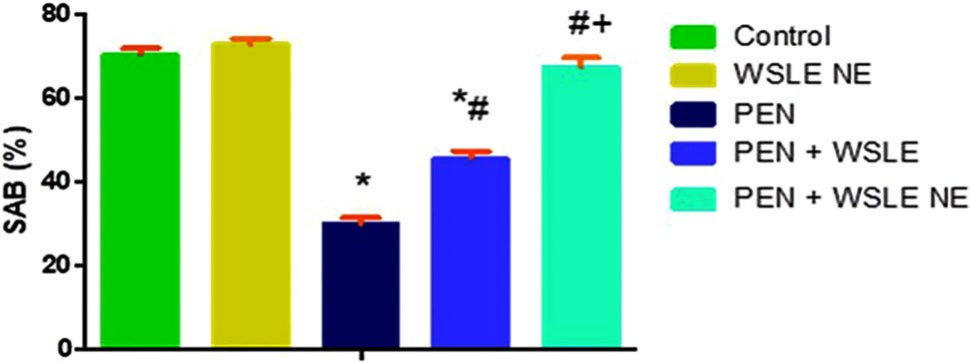
WSLE NE significantly alleviated PEN-induced memory dysfunction in rats through improving spontaneous alteration behavior (SAB) percentage in Y- maze test. Values expressed as mean ± SD (*n* = 6) and analyzed via one-way ANOVA and Tukey’s test. *, #, + represent significant values when compared to control, PEN-treated and WSLE-treated groups, respectively at *P* ≤ 0.01

The Y-maze test was conducted to assess the working memory in rats following PEN treatment. The results of the Y-maze test exhibited that SAB was significantly affected following PEN exposure [*F* (4, 25) = 737.804, *p* < 0.01]. Tukey’s post-hoc test emphasized that PEN-exposed rats revealed a significantly lower SAB compared to the control (*P* ≤ 0.01). However, WSLE NE-treated rats showed enhanced SAB compared to PEN-exposed rats (*P* ≤ 0.01). A more significant improvement in SAB was observed following treatment with WSLE NE than the extract alone (Fig. 5).

### Hot plate test

**Fig. 6:**
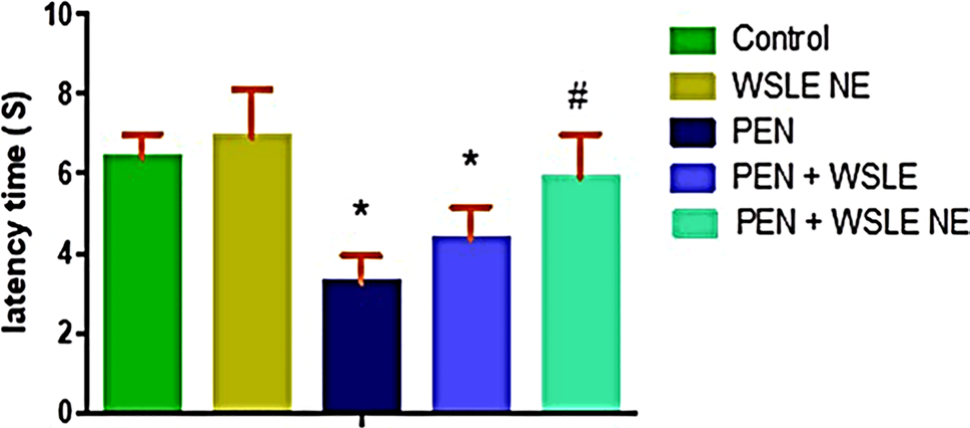
WSLE NE ameliorated pain sensation and neuropathic pain induced by PEN through increasing the latency time (S) in hot plate test. Values expressed as mean ± SD (*n* = 6) and analyzed via one-way ANOVA and Tukey’s test. *, #, + represent significant values in contrast to control, PEN-treated and WSLE-treated groups, respectively at *P* < 0.01

This test was used to evaluate the pain sensation and nociceptive response in rats. Results obtained from the hot plate tests (HPT) revealed that the latency time, elapsed between contact of rats to the hot plate surface till reaction, and tolerance to heat were significantly affected following PEN treatment [*F* (4, 25) = 17.612, *P* < 0.01]. Tukey’s posthoc test revealed that rats were extremely sensitive to heat following PEN treatment with a notable reduction in the latency time (*P* ≤ 0.01) in comparison to the control group (Fig. 6). However, WSLE NE-gavaged animals displayed a significant increase in the latency time and tolerance to the radiant heat in contrast to PEN-exposed rats (*P* < 0.01). A more significant increase in latency time was observed following treatment with WSLE NE compared to WSLE.

### Acetylcholinesterase (AChE) activity

**Fig. 7:**
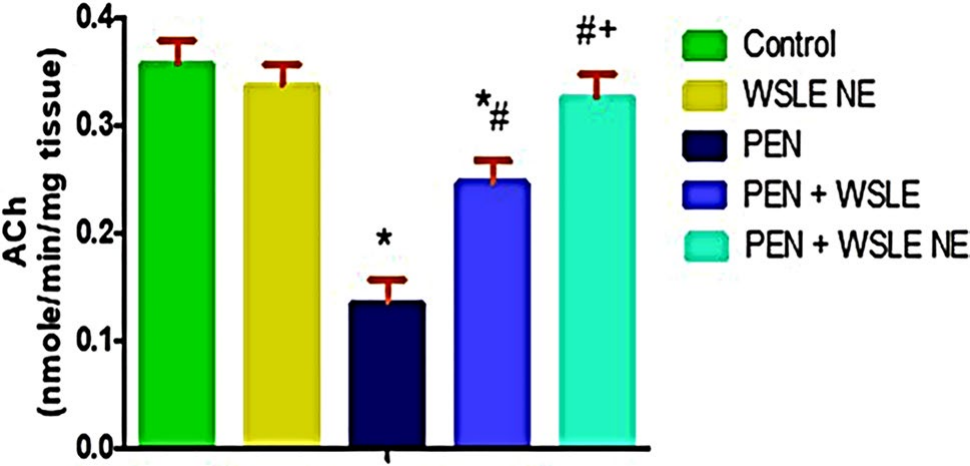
WSLE NE-treated group significantly increased AChE activity in brain tissues of rats following exposure to PEN. Data expressed as mean ± SD (*n* = 6) and analyzed via one-way ANOVA and Tukey’s test. *, #, + represent significant values in contrast to control, PEN-treated and WSLE-treated groups, respectively at *P* < 0.01

A significant effect of PEN treatment on AChE activity in brain tissues was revealed by one-way ANOVA [*F* (4, 25) = 114.392, *p* ≤ 0.01]. Tukey’s post-hoc test exhibited that PEN exposure considerably lowered AChE activity (*P* ≤ 0.01) in contrast to the control group. However, the WSLE NE-gavaged group substantially enhanced AChE activity (*P* ≤ 0.01) compared to the PEN group. Furthermore, WSLE NE-treated group considerably (*P* ≤ 0.01) boosted AChE activity as compared to WSLE-treated group (Fig. 7).

### WSLE NE alleviated the inflammatory response induced by PEN

**Fig. 8:**
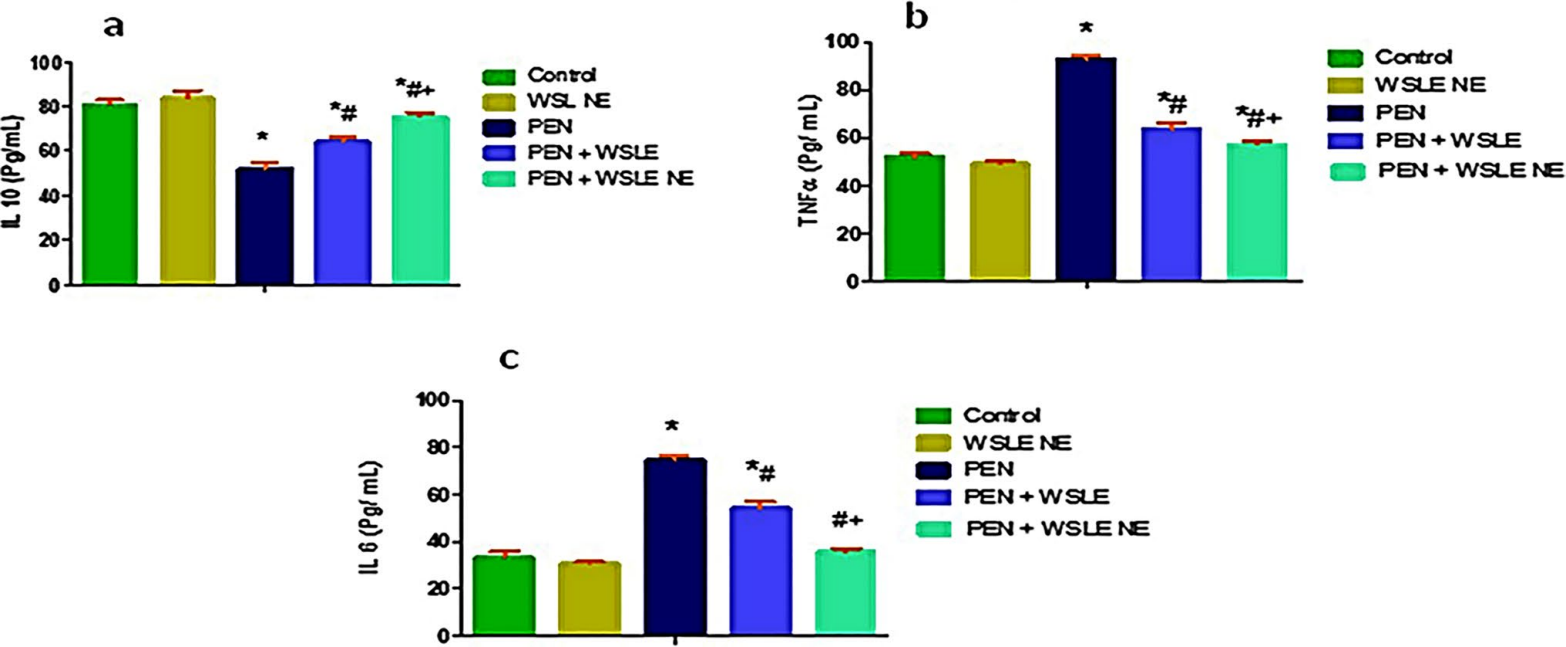
WSLE NE significantly modulate inflammatory cytokines including **a** TNFα, **b** IL6 and (**c**) IL10 in brain tissue of rats after exposure to PEN. Data expressed as mean ± SD (*n* = 6) and analyzed via one-way ANOVA then Tukey’s test. *, #, + represent significant values when compared to control, PEN-treated and WSLE-treated groups, respectively at *p* < 0.01

Inflammatory cytokines including IL-10, TNF-α and IL-6 were quantified in brain tissues. Results revealed a marked impact of PEN exposure on cytokines levels as IL-10 [*F* (4, 25) = 173.576, *p* < 0.01], TNF-α [*F* (4, 25) = 598.018, *p* < 0.01], and IL-6 [*F* (4, 32) = 557.675, *p* < 0.01]. The PEN-treated group exhibited a significant rise in TNF-α and IL-6 with a notable decrease in IL-10 (*p* < 0.01) in contrast to the control group. Meanwhile, both WSLE and WSLE NE- treated groups showed significant reduction in TNF-α and IL-6, in addition to elevation of IL-10 compared to the PEN-treated group (*p* < 0.01). Furthermore, the WSLE NE-treated group exhibited more beneficial benefits in the reduction of proinflammatory cytokines as compared to the WSLE-treated group (Fig. 8).

### WSLE NE reduced PEN-induced oxidative damage

**Fig. 9:**
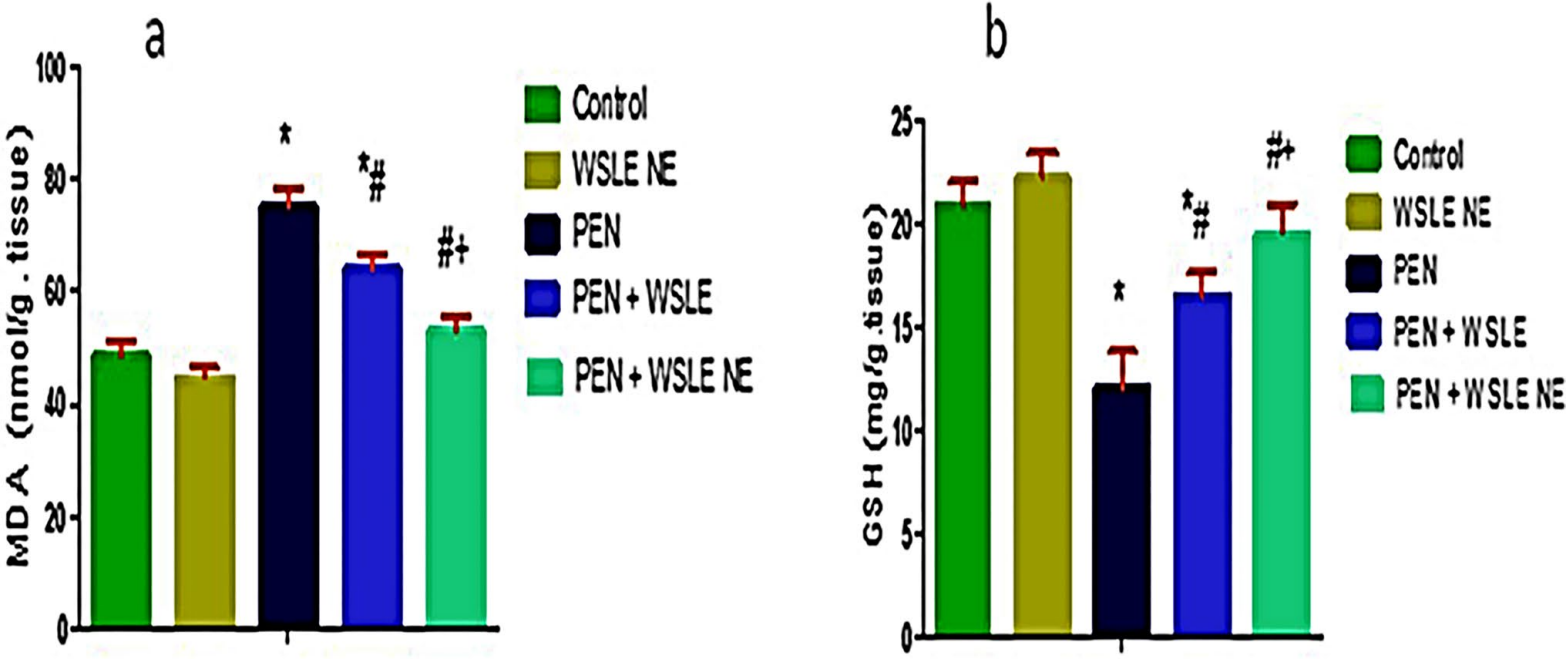
WSLE NE markedly protects against oxidative damage in brain tissue of rats exposed to PEN through regulating oxidative biomarkers including **a** MDA and **b** GSH. Values expressed as mean ± SD (*n* = 6). Data was analyzed via one-way ANOVA and Tukey’s test. *, #, + represent significant values when compared to control, PEN-treated and WSLE-treated groups, respectively at *P* ≤ 0.01

**Fig. 10:**
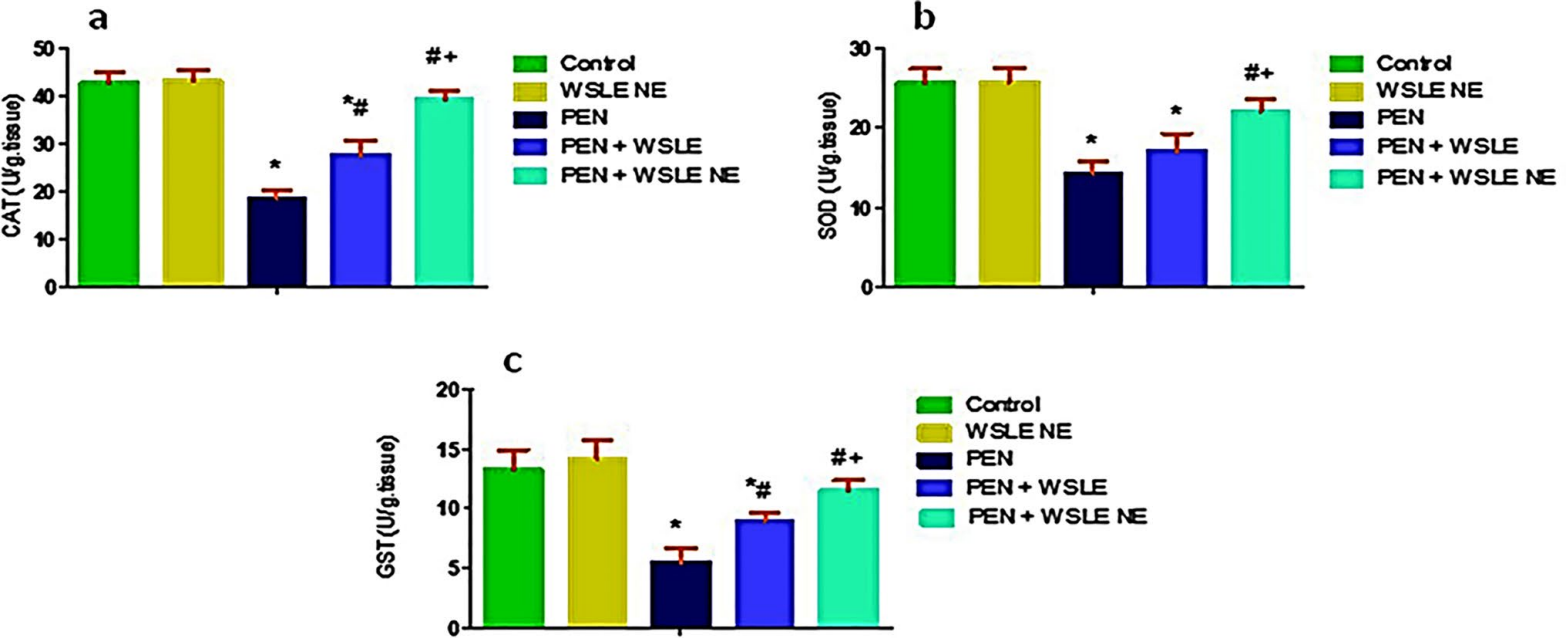
WSLE NE significantly enhanced the antioxidant enzymes levels composing **a** CAT, **b** SOD and (**c**) GST in brain tissue of rats exposed to PEN. Values expressed as mean ± SD (*n* = 6). Data was analyzed via one-way ANOVA then Tukey’s test. *, #, + represent significant values when compared to control, PEN-treated and WSLE-treated groups, respectively at *P* ≤ 0.01

GSH and MDA were evaluated in brain tissue samples and results demonstrated a substantial effect of PEN treatment on GSH [F (4, 25) = 55.149, *p* < 0.01] and MDA [*F* (4, 25) = 143.576, *P* < 0.01]. PEN-exposed group exhibited a substantial reduction in GSH levels (*P* < 0.01) with a noticeable rise in MDA levels (*p* < 0.01) in contrast to the control group. Meanwhile, compared to PEN-exposed rats, the WSLE NE-treated group substantially boosted GSH (*p* ≤ 0.01) and lowered MDA levels (*P* ≤ 0.01). In comparison to WSLE-treated group, WSLE NE markedly increased GSH (*P* ≤ 0.01) and declined MDA levels (*P* ≤ 0.01) (Fig. 9). Antioxidant enzymes such as CAT, GST, and SOD were also assessed (Fig. 10). Data displayed a notable impact of PEN treatment on CAT [*F* (4, 25) = 139.604, *P* ≤ 0.01], GST [*F* (4, 25) = 48.801, *P* ≤ 0.01], and SOD [*F* (4, 25) = 50.399, *P* ≤ 0.01]. Post-hoc analysis showed that CAT, GST and SOD activities were substantially lowered following PEN administration in contrast to control animals (*P* ≤ 0.01). Fortunately, both WSLE and WSLE NE-gavaged groups revealed a marked rise in CAT, GST and SOD activities in contrast to PEN-exposed group (*P* ≤ 0.01).

## Quantitative real-time PCR

**Fig. 11:**
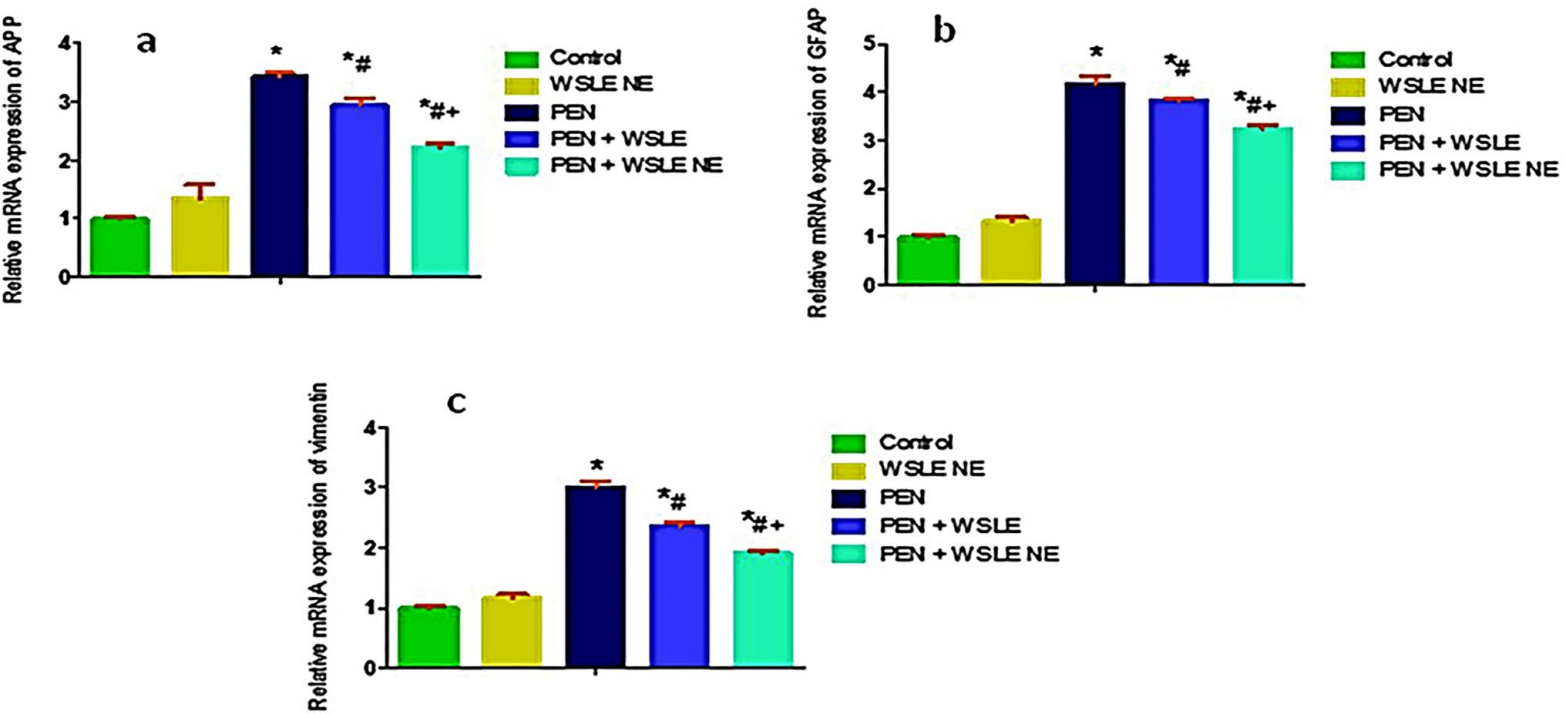
WSLE NE significantly regulated the molecular expression of **a** APP, **b** GFAP, and **c** vimentin in brain tissue of PEN-induced neurotoxicity models. Data expressed as mean ± SD (*n* = 6) and analyzed via one-way ANOVA and Tukey’s test. *, #, + represent significant values when compared to control, PEN-treated and WSLE-treated groups, respectively at *P* < 0.01

**Fig. 12:**
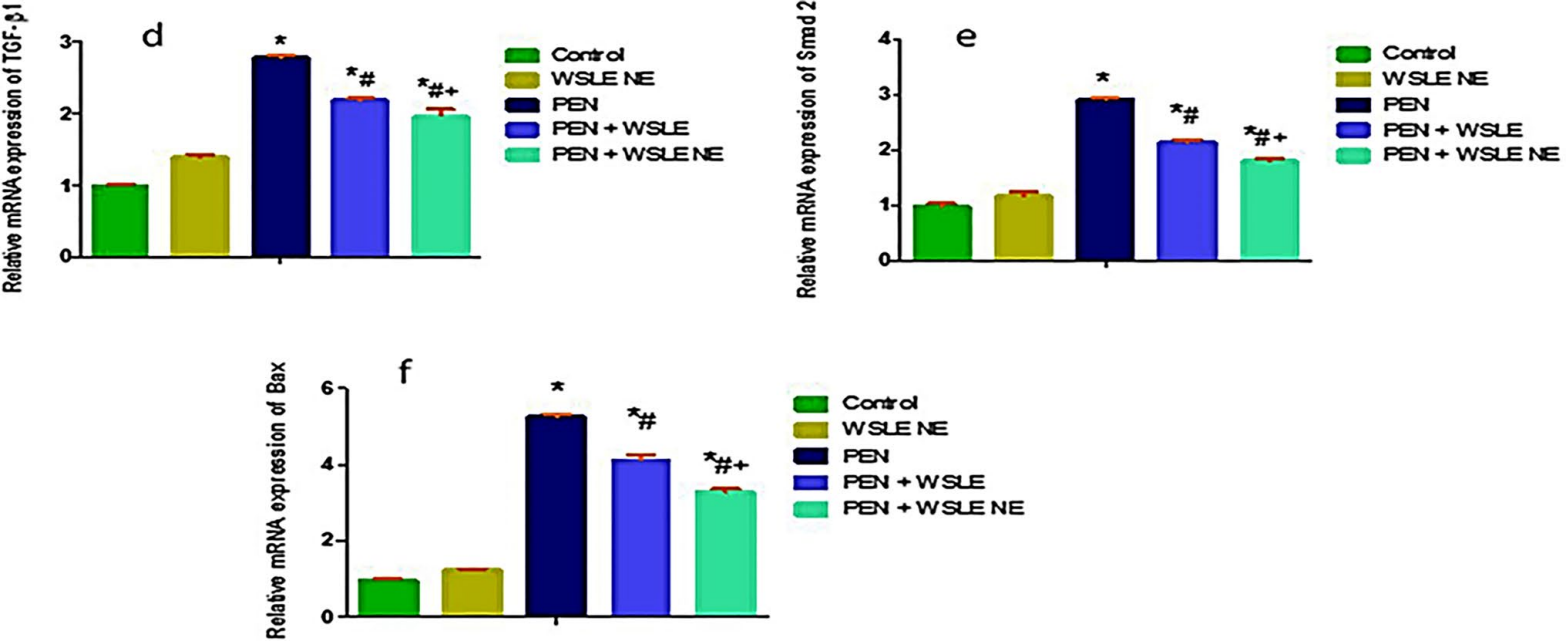
WSLE NE significantly regulated the molecular expression of **d** TGF-β, **f** Smad2, and **e** Bax genes in brain tissue of rats were measured. Data expressed as mean ± SD (*n* = 6) and analyzed via one-way ANOVA then Tukey’s test. *, #, + represent significant values when compared to control, PEN-treated and WSLE-treated groups, respectively at *P* < 0.01

The relative mRNA expression of APP, GFAP, vimentin, TGF-β1, Smad2 and BAX genes were measured in rats' brain tissue (Figs. 11,12). The results showed that the expression level of APP, GFAP, vimentin, TGF-β1, Smad2, and BAX genes was substantially elevated in the PEN-treated group when compared with the control group (*P* ≤ 0.01). On the other side, these genes expressions level were significantly downregulated following WSLE NE treatment when compared to PEN-exposed group (*P* ≤ 0.01).

## Histopathological and immunohistochemical evaluation

**Fig. 13:**
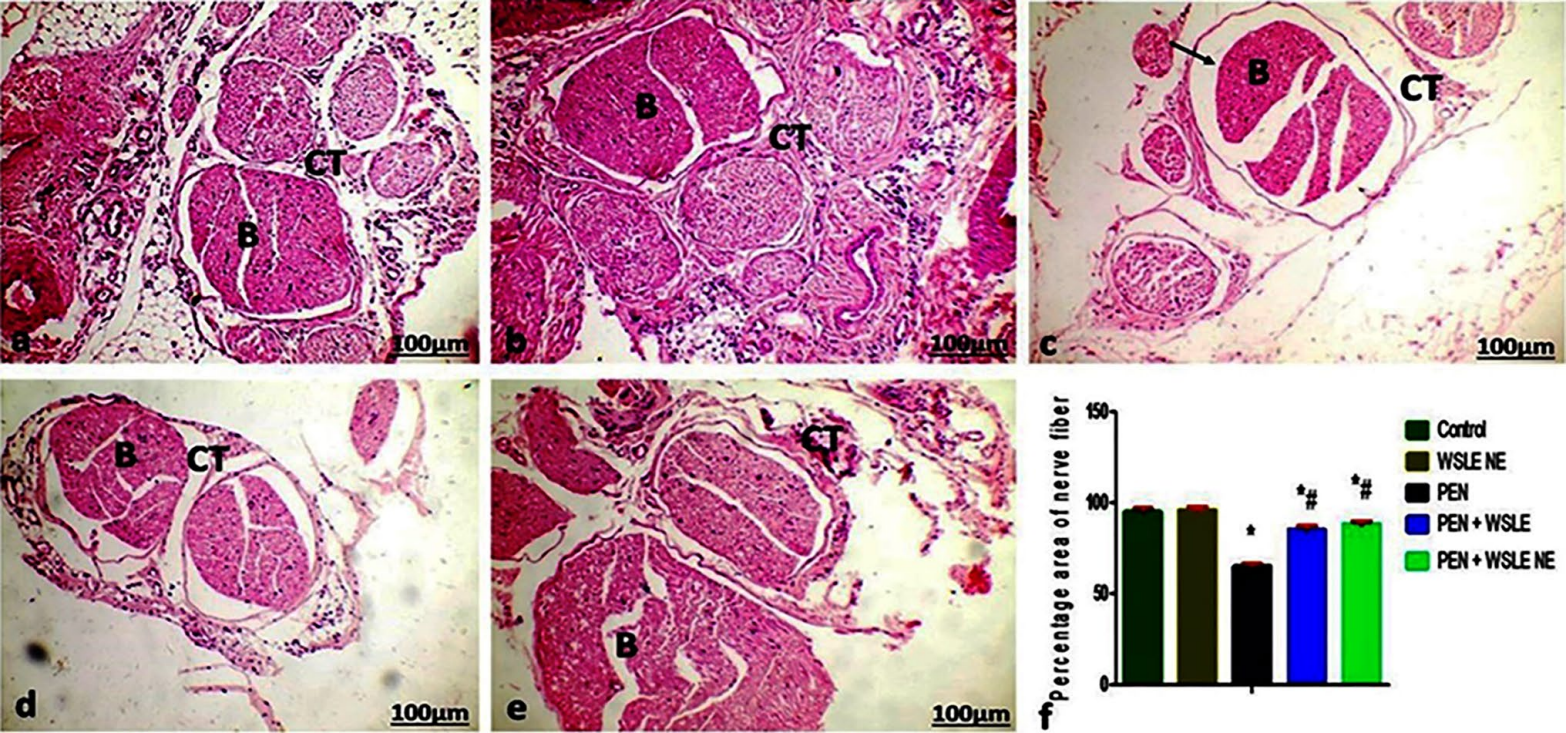
Light Photomicrographs showed histological examination of sciatic nerve of **a** control, **b** WSEL NE, **c** PEN **d** WSEL + PEN **e** WSEL NE + PEN groups (original magnification: × 100, bar: 100 μm). Arrow: presented the decrease of nerve fiber. B: bundle of nerve fiber and CT: connective tissue **F** Analysis of percentage area photomicrographs of the nerve fiber. Data are expressed as a percentage of the nerve fiber (*n* = 6). *, #, + represented significant values at (*P* ≤ 0.01) analyzed via one-way ANOVA and Tukey’s test

The sciatic nerve in control and WSEL NE- treated groups showed normal nerve fiber bundles. Each nerve fiber, comprising the Schwann cells, is surrounded by endoneurium that composed of areolar connective tissues carrying blood vessels. Each nerve fiber bundle is enclosed by perineurium (Fig. 13a, b). PEN-treated group showed marked decrease in the nerve fiber bundles when compared with the control group (Fig.13 c). WSEL + PEN and WSEL NE + PEN-treated groups showed normal histological structure as control (Fig.13 d, e).

Histomorphometric evaluation of the sciatic nerve supported the histologic findings observed in the different groups (Fig. 13 f). Comparing the regular distribution of the nerve fibers in normal nerves with the PEN treated group, the nerves cross-sections were dominated by a small percentage area of the nerve fibers. Increasing numbers of percentage areas of the nerve fibers were observed in the WSLE group and WSLE NE-treated groups.

**Fig. 14:**
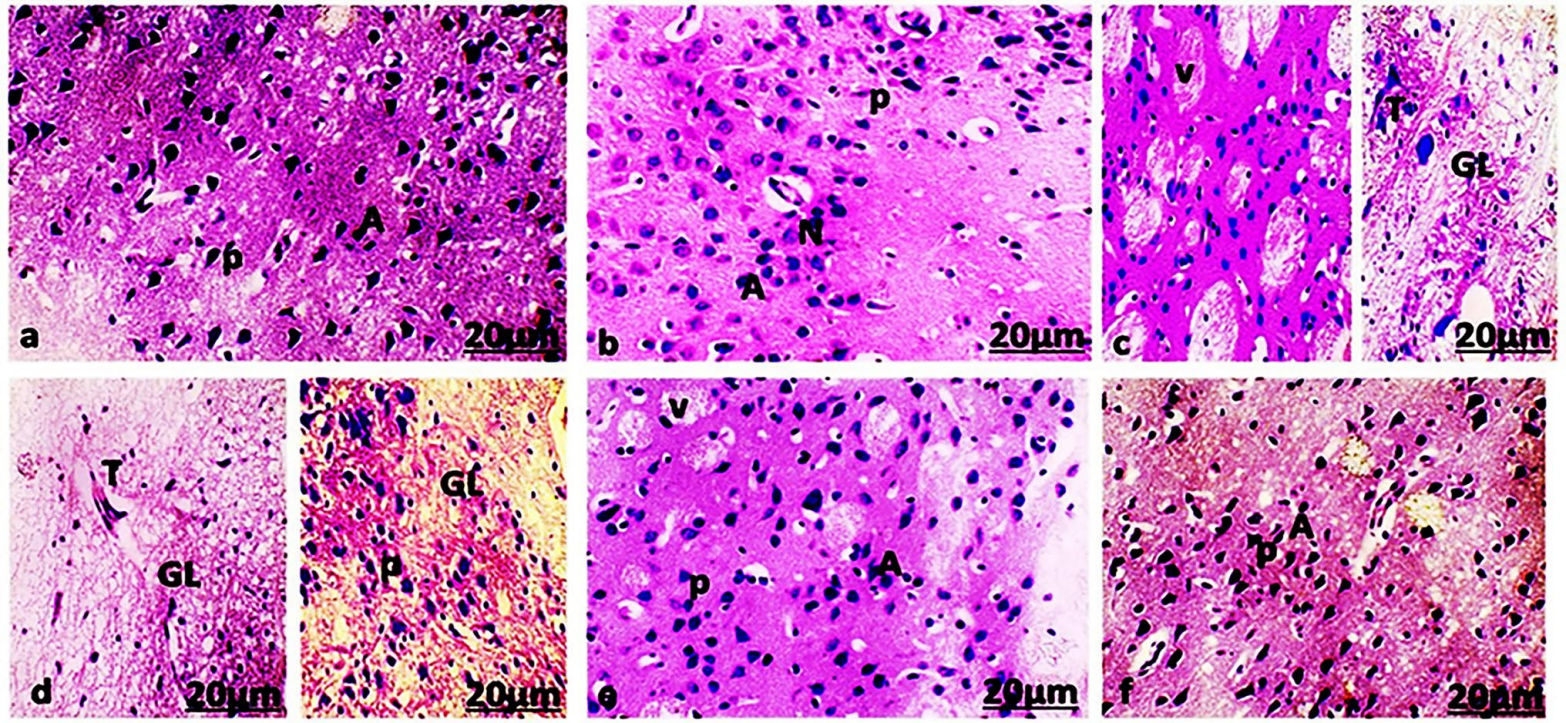
Light Photomicrographs showed histological examination of cerbral cortex of **a** control, **b** WSLE NE, **c **and **d** PEN **e** WSLE + PEN **f** WSEL NE + PEN groups (original magnification: × 400, bar: 20 μm). P; pyramidal cells, A; astrocytes, N; neuroglial cells, V; vacuoles, T; Neurofibrillary tangles, GL; gliosis

Cerebral sections in control and WSELE NE-treated groups showed normal histological appearance with normal pyramidal and astrocytes in addition to neuroglial cells (Fig. 14a, b). Meanwhile, cerebral sections of the PEN-exposed group displayed histological alteration associated with vacuolation in neurocytes, edema, gliosis and neurofibrillary tangles (Fig. 14c, d). The WSLE cotreated with PEN group showed normal histological structure and architecture of the cerebral cortex with mild vacuolation (Fig. 14 e). The WSLE NE cotreated with PEN group showed normal histological structure and architecture with pyramidal cells, astrocytes, and neuroglial cells (Fig. 14 f).

## Immunohistochemical assessment

**Fig. 15:**
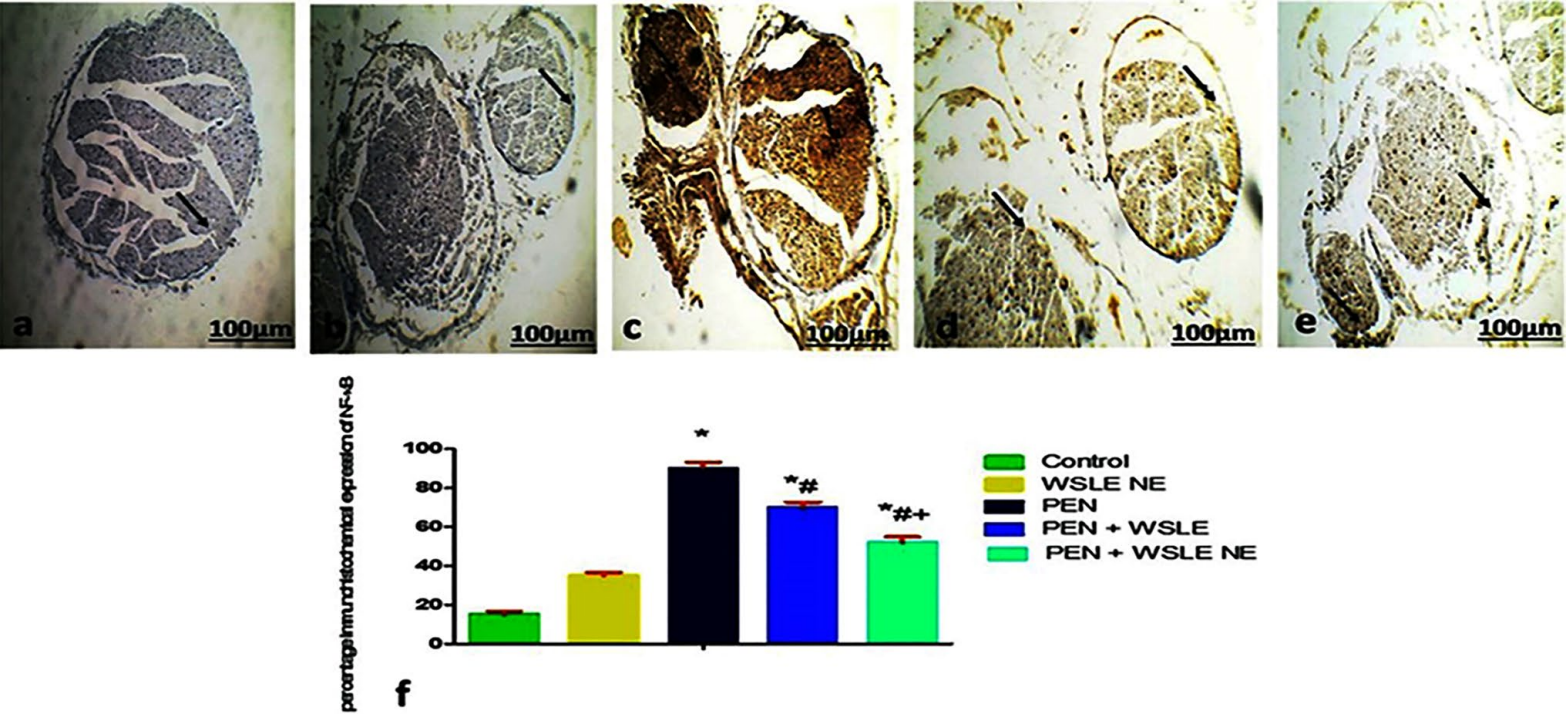
Light Photomicrographs displayed NFκB immunohistochemical expression in sciatic nerve of **a** control, **b** WSEL NE, **c** PEN **d** WSLE + PEN **e** WSLE NE + PEN groups (original magnification: × 10, bar: 100 μm). The positive staining of is displayed by a brown color of cytoplasm (arrows). **F**. Data presented as a percentage of total staining tissue area (*n* = 6). Analysis of NFκB immunohistochemical photomicrographs was assessed. *, #, + represented significant as at (*P* ≤ 0.01) analyzed via one-way ANOVA and Tukey’s test

**Fig. 16:**
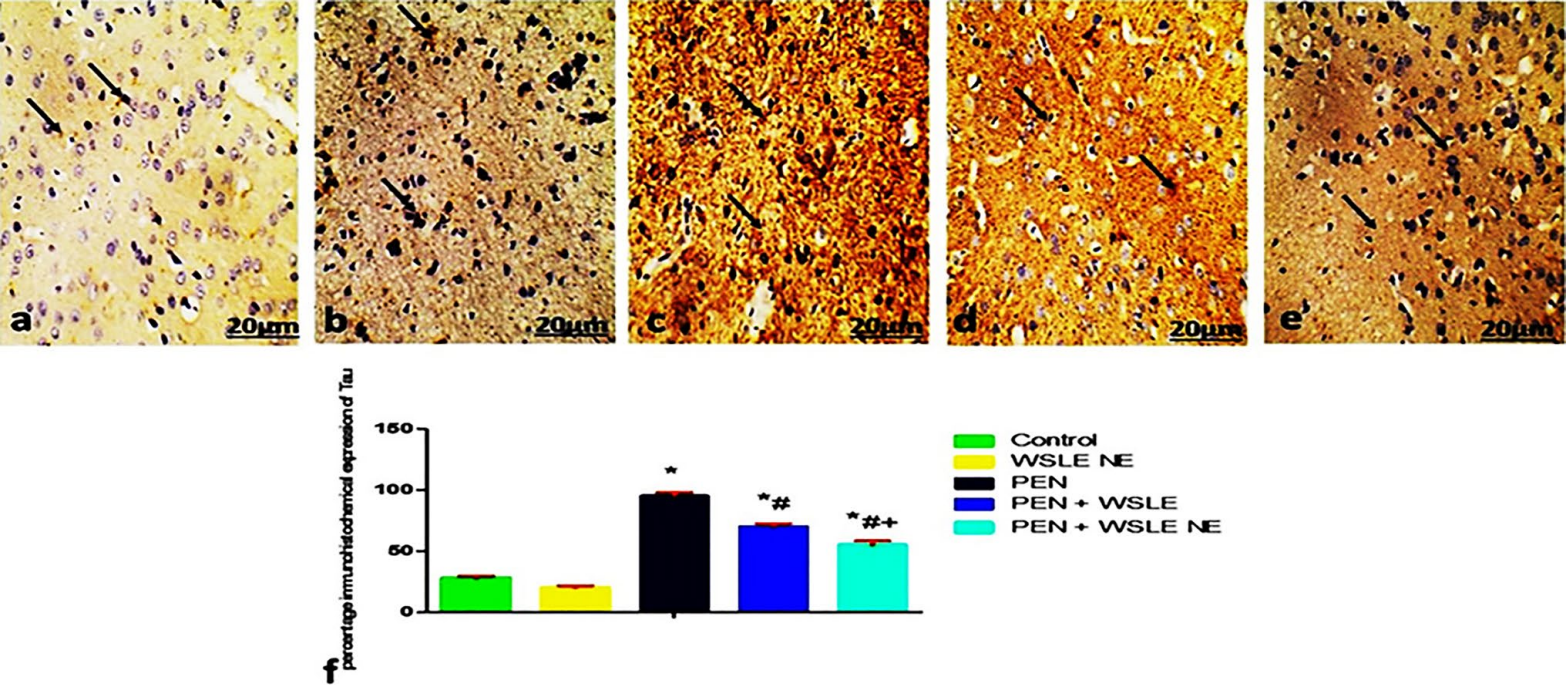
Light Photomicrographs display Tau immunohistochemical expression in cerebral cortex of **a** control, **b** WSLE NE, **c** PEN **d** WSLE + PEN **e** WSLE NE + PEN groups (original magnification: × 400, bar: 20 μm). The positive staining of Tau is presented by a brown color of cytoplasm (arrows). **F** Data presented as a percentage of total staining tissue area (*n* = 6). Analysis of Tau immunohistochemical photomicrographs was assessed between groups. *, #, + represented significant as at (*P* ≤ 0.01) analyzed via one-way ANOVA and Tukey’s test

Figures 15 and 16 showed the immunohistochemical staining of NF-κB in the sciatic nerve section and Tau in the cerebral cortex section, respectively. Administration of PEN increased the NF-κB expression in the sciatic nerve section Fig. 14 c and Tau in the cerebral cortex section (Fig. 16 c). At the same time, WSLE and WSELE NE-treated groups lowered the degree of positive staining for NF-κB in the sciatic nerve section and Tau in the cerebral cortex section compared to the PEN-exposed group. The histograms of the quantitative analysis assessed by percentage area of staining method using Fiji ImageJ software are shown in Fig. 15 d and 16 d, respectively.

## Discussion

PEN, a triazole fungicide, act through impairment of the sterol synthesis with excessive production of free radicals leading to change in membrane permeability, vacuolization, disintegration, and cell injury (El-Shershaby et al. 2020b). Moreover, it could pass through the neuronal membrane due to its lipophilic nature causing histopathological changes in brain tissues besides impairment of the cholinergic function and behavioral activity (Chaâbane et al. 2018).

*W. somnifera* in either raw form or their leaves extract utilized as complementary medicine in neurological disorders (Paul et al. 2021b). The current study was undertaken for phytoconstituents estimation and evaluating the chemical compositions of WSLE since knowledge of the plant phytoconstituents will be important in various pharmacological studies (Dutra et al. 2016). Hence, there was a necessitate need to provide LC–MS/MS studies in that plant extract. LC–MS/MS analysis of WSLE revealed various phytochemical components such as flavonoids, phenolic acids and withanolides. Some of these components could suppress neuroinflammation and neurodegenerative disorders. Previous studies displayed that some flavonoids and phenolic acids detected in this study including luteolin, kaempferol, quercetin, caffeic acid and trans-cinnamic acid revealed neuroprotective effect through various mechanisms with potent antioxidant and anti-inflammatory effects (Chandra et al. 2019; Chang et al. 2022; Fernandes et al. 2023; Kempuraj et al. 2021; Wang et al. 2022). Furthermore, withanolides which are major bioactive components of *W. somnifera* as withanolide-A exert neuroprotective ability through suppression of pro-inflammatory factors and oxidative damage (Zhu et al. 2020), modulation of neural chemical mediators like acetylcholine (Akhoon et al. 2016) and modification of Aβ processing (Pandey et al. 2018).

Research efforts have been developed to improve therapies for neurological disorders (Zhang et al. 2018). The inability of most neuroactive drugs to reach brain parenchyma in sufficient amounts hinder their role in treating various neurological disorders (Furtado et al. 2018). Despite this worrying picture, substantial evolution of innovative drug delivery systems in CNS, including nanoemulsions, has recently become.

necessary. Nanoemulsions are effectively synthesized through various techniques such as the high energy emulsification method proposed in this study (Singh et al. 2017). The formulated nanoemulsion was characterized using DLS and revealed homogenous and stable narrow-sized droplets.

with particle size ranging from 30 to 50 nm which within the suitable range to cross the blood–brain barrier (BBB) (Thuraisingam et al. 2022). Furthermore, the PDI of the formulated nanoemulsion was 0.12, which reflected homogeneity of the nanoemulsion within a good monodisperse system (Badawy et al. 2018). TEM analysis revealed spherical and homogenous nanodroplets (30–50 nm), which is comparatively lesser than DLS measurement since TEM analyzes the sample in its dried state, giving the real actual size morphology of the sample (Ghazy et al. 2021a). The stability of the nanoemulsion is associated with the usage of the ultrasonication technique which generates high-intensity ultrasonic waves creating intense disruptive forces required to fracture water and oil phases into nano-sized droplets (Niknam et al. 2020). The nanodroplets are further stabilized through the surfactant and cosurfactant molecules, which reduce the extent of collision and agglomeration of the nanoemulsion via stearic hindrance (Roy and Guha 2018).

The present study revealed that PEN administration resulted in marked disruption in neurobehavioral parameters, oxidative stress biomarkers, inflammatory cytokines, and cholinergic functions which proves its direct effects on CNS. These changes were significantly ameliorated by WSLE NE since *W. somnifera* is a potent herb that used to treat a variety of diseases in traditional Indian medicine (Prakash et al. 2014b). Various methods designed for neurobehavioral assessment of neurotoxic agents are based on changes in learning, motor function, sensory function, and natural occurring behaviors. Open field and forced swimming tests were employed for the assessment of anxiety and behavioral despair levels, respectively, in treated animals. Our findings revealed that PEN administration evoked significant anxiogenic effect as indicated in OFT by reduction in time spent in the inner zone besides rearing and grooming frequency, moreover depressive-like behavior was quite evident in treated groups, as indicated by the increased immobility time in FST, suggesting emotional stress and major depressive disorders. These findings are corroborated by previous studies (El-Shershaby et al. 2020b; Morgan et al. 2021b), which emphasized that this anxiogenic effect may attributed to degeneration of the dopaminergic neurons in the substantia nigra region of the brain with functional impairment (Liu et al. 2022b). WSLE NE markedly reversed these changes mainly through the antioxidant effect of glycowithanolides, an active constituent of *W. somnifera* (Mohanty et al. 2016; Sankar et al. 2007). Furthermore, WSLE NE displayed differential neuroprotective effects as compared with free extract due to the lipophilic nature of lipid-based NPs that allow them to cross the BBB efficiently with high bioavailability, biocompatibility, biodegradability, and drug-loading efficiencies (Ramires Júnior et al. 2021). The Y-maze is a specific task for working memory (Lainiola et al. 2014) or spatial recognition in rodents (Abdulbasit et al. 2018). present experiments investigated the effects of WSLE or WSLE NE on PEN-associated memory disorders. The protective effect of WSLE or WSLE NE against the learning and memory decline in PEN-treated rats has been established. The learning and memory disorder of PEN-treated rats is associated with the histological damage of brain cerebral cortex which according to Myhrer (2003) is believed to trigger neurotransmission alterations and even learning and memory interferences. In agreement with previous studies, PEN administration impaired the spatial memory with significantly reduced spontaneous alternation behavior (El-Shershaby et al. 2020b; Jia et al. 2020b).This result also indicated that WSLE or WSLE NE improved the PEN working memory disorders. The cotreatment of rats with WSLE NE markedly improved the working memory, probably due to its cholinomimetic activity (Choudhary et al. 2017), which was verified by the increase in spontaneous alternation percentage as compared to PEN-treated rats. Moreover, administration of the extract nanoemulsion displayed more beneficial effects on cognition than the extract alone, presumably due to the improvement of the bioavailability of active constituents of the extract (Ghazy et al. 2021b).

Peripheral neuropathy is generally characterized by increased sensitivity to pain (thermal hyperalgesia) mainly due to deterioration of the sciatic nerve (Liu et al. 2021). The present study displayed that PEN significantly elevated thermal hyperalgesia observed by the decrease in reaction time in the hot plate test as reported previously (Liu et al. 2022a; Morgan et al. 2021b). Moreover, PEN induced injury and degeneration of nerve fiber in the sciatic nerve tissue. The damage to sciatic nerve induced by PEN, according to Vincent et al. (2004) may be due to the increased oxidative damage, which develops hypoxia with subsequent damage to the blood vessel supplies the peripheral nerve, or according to Gupta and Steward (2003) may be due to the increased water influx in the Schwann cells lead to degeneration of the nerve cells. However, WSLE NE, based on the present findings, can attenuate neuropathic pain in PEN-treated animals confirming the antinociceptive or analgesic effect of the extract nanoemulsion which may be due to its ability to suppress free radicals and proinflammatory mediators (Akbar et al. 2020; Orrù et al. 2014; Srivastav and Das 2014) and protect nerve fiber of the sciatic nerve from the damage. This study provided convincing evidence that WSLE NE treatment attenuated PEN induced neuropathic by decreasing the NF-κB immunohistochemical expression in the sciatic nerve in comparison to the induced by PEN. Based on both the behavioral, histological, and immunohistochemical expression outcomes, the most interesting finding is that WSLE NE treatment possesses potential benefits in improving behavioral tests and reducing the histomorphological damage of sciatic nerve.

AChE, a vital enzyme incorporated into the cholinergic function, is an essential regulator of the neurobehavioral processes (Mani et al. 2014); in the current study, PEN-treated animals exhibited a marked decline in the AChE activity as compared with the control group that may be due to interaction with the esteratic sub-region of AChE with subsequent prevention of the substrate from binding to the enzyme or excessive ROS production as reported previously (Alkan Uçkun et al. 2020; Jia et al. 2020b) and as found in the present study. Furthermore, impairment in cognitive function, locomotor and exploratory activities observed in the PEN-exposed rats may be related to cholinergic dysfunction (Adedara et al. 2018). Meanwhile, the enhancement in the cholinergic neurotransmission and neurobehavioral parameters in rats co-treated with WSLE NE was evidenced by the elevation in AChE activity.

Neuroinflammation is inevitably linked in the pathogenesis of various neurodegenerative disorders (Obrador et al. 2020). Pro-inflammatory cytokines such as TNF-α, and IL-6 can Immediately trigger inflammatory and apoptotic pathways, resulting in loss of neuronal activity and neurodegeneration (Jamwal et al. 2015; Rajput et al. 2017). In the current study, treatment with PEN provoked significant elevation of pro-inflammatory IL-6 and TNF-α levels in brain tissue. At the same time, it markedly reduced the anti-inflammatory IL-10 level which is quite similar to previous studies (Chaâbane et al. 2018, 2015), whereas treatment with WSLE NE significantly alleviated the alteration in these cytokines levels that may also attributed to the glycowithanolides content and the higher antioxidant activity of the nanoemulsion compared to the extract leading to prevention of neuroinflammation and apoptosis (Chandra et al. 2012; Gupta and Singh, 2014).

Oxidative stress may occur when production of reactive oxygen species exceeded the antioxidant defense systems (Dhouib et al. 2017). MDA is a reliable indicator of free radical formation in the biological systems that aggravate disruption of the antioxidant enzymes activity (Singh and Kumar, 2019). Our results revealed a significant increase in MDA levels in PEN-treated rats when compared to control, while cotreatment with WSLE NE markedly ameliorated MDA to the normal levels. This finding is in good agreement with previous reports, which explained the protective effects of WSLE against induced oxidative damage could either be directly via scavenging of free radicals and inhibiting peroxidation of lipids or indirectly through the enhancement of the antioxidant enzyme activities (El-Sabbagh et al. 2022; Singh et al. 2020).

GSH, a non-enzymatic antioxidant, combines electrophilic compounds under the influence of GST (Baldissera et al. 2021). SOD and CAT, enzymatic antioxidants, constitute the most important defense mechanisms against oxidative stress or toxic effects of oxygen metabolism (Kamboj et al. 2006). In the present investigation, a marked decrease in GSH, GST, SOD and CAT activities in rats exposed to PEN as compared to the control group. Our results are in line with previous, which reported that a decrease in the antioxidant enzyme system might be due to a response to ROS overproduction and the antioxidant capacity was exceeded by the amount of the free radicals generated (Chaâbane et al.^2016^, 2017c). On the other hand, WSLE NE considerably enhanced both enzymatic and non-enzymatic antioxidants levels in the brain. This antioxidant property was attributed to the active constituents of *W. somnifera* including Sitoindosides VII-X and Withaferin A (Glycowithanolides) besides the presence of other antioxidant compounds such as flavonoids and polyphenols (Visavadiya and Narasimhacharya 2007). Moreover, the obtained droplet size of the extract nanoemulsion suggested that it can easily permeate through membrane lipid bilayer due to its smaller size so it could neutralize free radicals more efficiently, thereby enhancing its antioxidant activity (Sharma et al. 2019).

To dissect underlying molecular mechanisms of PEN-induced neurotoxicity, TGF-β/Smad signaling pathway was suggested to be as a possible target of PEN in brain tissues. TGF-βs are superfamily of multifunctional cytokines, including TGF-β1, 2, and 3, and represent neurotrophic factors that are involved in the cellular response to injury and play a key role in astrocyte reactivity, initiating and maintaining brain homeostasis, neuronal differentiation, and synaptic plasticity (Diniz et al. 2019; Schlecht et al. 2021). The altered TGF-β signaling pathway in glial cells contributes to the pathogenesis of various neurological diseases including vascular dementia, Alzheimer's disease, and Addison's disease (Krieglstein et al. 2011). TGF-β isoforms are secreted in a latent form and then activated through various cell surface integrins, proteases, and ROS in response to cellular injury before binding to its receptors (Kandasamy et al. 2020). Upon binding of TGF-β to its receptors, including receptor types 1 and 2, forms a heteromeric complex which phosphorylate and activate SMAD2 and SMAD3 to form complexes with SMAD4 and moves to the nucleus where they manage the transcription of TGF-β/Smad-responsive genes through binding with Smad binding element (SBE) (Kandasamy et al. 2014; Li et al. 2012). TGF-βs are widely expressed among various cell types in CNS including astrocytes, and identified are as key regulators of astrocyte reactivity and modulators of intermediate filament protein expression (Karampetsou et al. 2022; Li et al. 2008). Upregulation of intermediate filaments as GFAP and vimentin is a hallmark of astrocytes reactivity which are protective in normal conditions but may become maladaptive, like peripheral inflammatory responses, causing tissue damage neurotoxicity under certain circumstances (Brandebura et al. 2023; Li et al. 2019). Therefore, aberrant TGF-β signaling pathway, either decrease or increase, could play a crucial role in the pathogenesis of neurodegenerative disorders (Hong et al. 2023). Previous reports revealed that overexpression of TGF-β1 (the most comprehensively studied TGF-βs) promotes β-amyloid deposition, Tau hyperphosphorylation and NFTs formation in brain tissues accompanied by reactive astrogliosis (Karampetsou et al. 2022; Lee et al. 2010; Russo and Wharton 2022). The astrocyte-targeted TGF-β1 overexpression drives APP overproduction in astrocytes and promotes Aβ accumulation suggesting an astrocyte-specific mechanism that impaired TGF-β/Smad signaling pathway leading to overactivation of microglia, Aβ deposition, and apoptosis (Diniz et al. 2019; Ongali et al. 2010). The present study revealed that PEN displayed overexpression of APP, GFAP, vimentin, TGF-β1, Smad2, and BAX target genes in rats brain tissue. These results come in agreement with previous studies which displayed various neurodegenerative disorders following exposure to PEN that may be related to its capacity to induce sever oxidative damage in brain tissue through overproduction of ROS which could impair TGF-β/Smad signaling pathway in astrocytes, trigger apoptosis and affect neuronal functions (Beshay et al. 2020; Chaâbane et al. 2017a; Jia et al. 2020a). Moreover, Morgan et al., (2021a, b) (Morgan et al. 2021a) displayed that PEN exposure could increase expression level of bax and GFAP in brain tissue since PEN could easily cross the neuronal membrane to initiate ROS generation in mitochondria with subsequent apoptosis (El-Shershaby et al. 2020a). On the other side, cotreatment with WSLE and WSLE NE revealed a signification downregulation of APP, GFAP, vimentin, TGF-β, Smad2, and BAX target genes which coincided with previous studies which highlighted the neuroprotective effect of WSLE in a variety of neurological disorders (Jindal et al. 2022; Sharma 2023; Syed et al. 2021b). Prakash et al., (2014a, b) (Prakash et al. 2014a) displayed that WSLE reduced expression level of GFAP and Bax in brain tissues of mice exposed to paraquat and maneb combination. Furthermore, Moustafa et al. (2022) reported that treatment with WSLE significantly reduced TGF-1β, and vimentin levels in rats exposed to ionizing radiation. The neuroprotective effect of WSLE could be attributed to its high withanolides and flavonoid content, which prevent lipid peroxidation and increase antioxidant enzyme activity levels (Paul et al. 2021a). Additionally, Mandlik and Namdeo (2021) depicted that Withanolide-A could restore synapses and regenerate neurites in severely injured neurons via decreasing glial activation, nuclear factor kappa phosphorylation and amyloid accumulation (Dutta et al. 2018; Gupta and Kaur 2016). Interestingly, WSLE NE favorable effects exceed that of WSLE alone which may be due to the enhanced availability of the phytochemical constituents in the plant extract with subsequent efficient antioxidant and anti-inflammatory effects.

The cerebral cortex of rat exposed to PEN showed gliosis which referred to the reactive astrocytic response to a brain injury (Garcia-Estrada et al. 1993). The mechanism of triggered gliosis was attributed to Pro-inflammatory cytokines and oxidative damage (Wang et al. 2021b) that found increased in the present study. Gliosis is accompanied by the higher generation of the intermediate filament including GFAP, nestin, and vimentin, which leads to greater and more highly condensed glial processes and fibers (Gomes et al. 1999). This agreement with the finding with the increased relative mRNA expression of APP, GFAP, vimentin, TGF-β1, Smad2 and BAX genes were measured in rats brain tissue after PEN treatment found in the present study. The cerebral cortex of rat treated with PEN showed Alzheimer marker pathology. Neurofibrillary tangles appeared in the cerebral cortex of rats treated with PEN with increased immunohistochemical expression of filamentous tau proteins which suggested marker of Alzheimer pathology (Guillozet-Bongaarts et al. 2005).

Treatment with WSLE alone decreased the brain injury induced by PEN treatment with only mild vacuolation in the cerebral cells that appear in the cerebral cortex's histological section and decreased the filamentous tau proteins' immunohistochemical expression. The neuroprotective of WSLE was previously indicated by improving the histopathological change of the cerebral cortex (Konar et al. 2011). Treatment with WSEL NE prevented brain injury as prevented the Gliosis, neurofibrillary tangles, edema and vacuoles from appearing in the histological section of the cerebral cortex and decreasing the immunohistochemical expression of filamentous tau proteins. Further, the neuroprotective of *W. somnifera *nanocapsules was previously indicated by improving the cerebral cortex's histopathological change (Khalil et al. 2023). The present findings confirmed that WSLE NE could enhance the permeability of the plant extract constituents through the blood brain barrier to boost its neuroprotective effect against PEN-induced neurotoxicity.

## Conclusion

In conclusion, based on the findings obtained in this study, PEN promoted neuroinflammation caused by ROS which in turn induced damage to the brain and peripheral sciatic nerve. PEN caused a sharp increase in the relative mRNA expression of APP, GFAP, vimentin, TGF-β1, Smad2 and Bax genes measured in rats' brain tissue. WSLE NE improved the learning and memory dysfunction and suppressed the inflammatory and stress damage of PEN-treated rats better than WSLE alone. These findings suggested that WSLE NE is a novel therapeutic approach for the treatment of cognitive impairments. WSLE NE markedly enhanced the permeability of plant extract constituents through the blood brain barrier to boost its neuroprotective effect against PEN-induced neurotoxicity.

## Data Availability

The authors confirm that the data supporting this study's findings are available within the article and its supplementary material.
